# Oral health of individuals with intellectual disabilities: a global cross-sectional study from the special olympics world games 2023

**DOI:** 10.1007/s00784-025-06331-3

**Published:** 2025-04-16

**Authors:** Marc Auerbacher, Susanne Krämer, Peter Schmidt, Andreas G. Schulte, Imke Kaschke, Falk Schwendicke, Luc Marks, Benedikt C. Spies, Anna-Lena Hillebrecht

**Affiliations:** 1 Department of Conservative Dentistry and Periodontology, University Hospital, LMU Munich, München, Germany; 2 Department of Dermatology, Medical Faculty and Medical Center, University of Freiburg, Freiburg, Germany; 3 Special Care Dentistry Unit, Facultad de Odontologia, Universidad de Chile, Santiago, Chile; 4 Department of Special Care Dentistry, Dental School, Witten/Herdecke University, Witten, Germany; 5 Special Olympics Germany, Berlin, Germany; 6 Faculty of Medicine & Health Sciences, University of Antwerp, Campus Drie Eiken, Universiteitsplein 1, Antwerp, 2610 Belgium; 7 Special Care in Dentistry, Department of Cranio-Maxillofacial Surgery, Antwerp University Hospital, 655 Drie Eikenstraat, Edegem, 2650 Belgium; 8 Department of Prosthetic Dentistry, Centre for Dental Medicine, Medical Centre, University of Freiburg, Freiburg, Germany; 9 Department of Prosthetic Dentistry, Faculty of Medicine, University of Freiburg, Hugstetterstr. 55, 79106 Freiburg, Germany

**Keywords:** Special needs, Athletes, Dental treatment needs, Oral disease, Intellectual disability

## Abstract

**Aim:**

The aim of this study was to analyze the interrelationships between oral health status and dental visits among athletes participating in the Special Olympics World Games Berlin, Germany 2023.

**Methods:**

This cross-sectional study was conducted within the framework of the Healthy Athletes Program at the Special Olympics World Games Berlin 2023, where 2109 athletes with intellectual disabilities (ID) from all over the world participated in voluntary oral health screenings. Data were collected through interviews and clinical oral examinations, following a standardized protocol. Statistical analyses included descriptive statistics, logistic regression models, and chi-square tests to assess regional disparities, with significance set at *p* < 0.05.

**Results:**

From a total of 7500 athletes that participated during the Special Olympics World Games 2109 athletes (28.1%) with intellectual disabilities from 152 countries participated in the oral health screening at the Special Olympics World Games Berlin 2023. Untreated caries were present in 40.6% of the athletes (*n* = 856). If the dentist was visited at least once a year neither the occurrence of untreated carious lesions (OR 1.02; *p* = 0.868) nor the rate of untreated lesions (OR 1.10, p-value 0.630), the number of missing teeth (OR 0.65) or gingivitis (OR 1.076) was significantly reduced.

**Conclusion /clinical relevance:**

This study highlights an overall insufficient oral health status in athletes with ID. Even those who visited a dentist within the last year, treatment needs were not reduced significantly. More efforts compensating reduced oral hygiene capabilities and optimizing a barrier-free provision of dental care are needed.

**Supplementary Information:**

The online version contains supplementary material available at 10.1007/s00784-025-06331-3.

## Introduction

According to the World Health Organization, there are approximately 200 million individuals with intellectual disability (ID) globally [1, 2]. The Convention on the Rights of People with Disabilities states that people with disabilities are entitled to receive care of the same quality and standard without discrimination as the rest of the population [3]. Furthermore, access to adequate medical care and nursing support is essential to enable people with disabilities to achieve the highest attainable standard of health. This comprehensive provision ensures not only their well-being but also their right to equal participation and inclusion in society. Although the life expectancy of people with ID has increased over the past decades as a result of advances in medicine, public health, education and technology, they continue to experience poorer health than the general population [4, 5].

The latter also affects oral health, including the ability to eat, speak and interact without embarrassment, discomfort or disease (oral-health-related-quality-of-life) [6–10]. Factors such as poor reflexes, motor impairments, high palate, maxillary hypoplasia, malocclusions, open bite and abnormalities in tooth morphology or eruptive pattern impact on oral health of individuals with ID [6, 11]. Individuals with ID have been reported to have poorer oral hygiene, higher plaque levels, more severe gingivitis and periodontitis than populations without ID. Moreover, individuals with some genetic disorders (e.g., Down syndrome) have higher risk of periodontal diseases, related to impaired immunologic responses [6, 12, 13]. Numerous individual, social and environmental factors influence oral care for individuals with ID. A coordinated organizational response is advocated involving collaboration between dental and intellectual disability services and training for caregivers and people with ID [14].

Access to dental examinations and treatments seems notably more challenging for individuals with ID as dental professionals are often not trained to treat this clientele [15, 16]. In order to maintain good health, people with ID need special oral health support programs tailored to their disability [17]. Most oral health care systems are based on demand for care and oral health care is provided by private dental practitioners to patients, with or without third-party payment system. There are still disparities in access to dental care for people with disabilities compared to the general population [18]. Some countries have organized public health services, providing oral health care, particularly for children and disadvantaged population groups [19]. As the group of people with ID is heterogeneous, there is little data on their access to oral health care and its effectiveness on the number of untreated caries lesions or the number of missing teeth in different regions of the world.

The Special Olympics Healthy Athletes Program has the primary goal of helping athletes with ID to improve their ability to train and compete in different sports programs of the Special Olympics. Special Smiles (SpecS) is the oral health component of this program. SpecS provides comprehensive oral health information to individuals, including dental screenings and instruction on proper oral health care interventions. An additional goal is to collect standardized data on oral health of individuals with disabilities. The resulting data has allowed to demonstrate that dental trauma and gingivitis are common in individuals with ID [20–22]. Moreover, oral health of athletes with ID seems poorer compared to athletes without ID [23]. In the context of the Special Olympics World Games Berlin, 2023, individuals with ID from across the world were assessed by trained and calibrated dentists and surveyed regarding their oral health behaviors and access to dental care. The present study aims to analyze the interrelationships between oral health status and dental visits among athletes with ID.

## Materials and methods

### Study design and participants

This cross-sectional study was conducted in 2023 within the framework of the Special Olympics World Games Healthy Athletes Program in Berlin, Germany. It is reported in accordance with the Strengthening the Reporting of Observational Studies in Epidemiology (STROBE) guidelines [24]. Ethical approval from the ethics committee of the Joint Ethical Committee of Charité Berlin (EA4/045/14) and informed consent were obtained.

### Setting

The Special Smiles ^®^ (SpecS) program included a registration/check-in station, a non-invasive dental screening station and a dental hygiene education station. At the dental screening station, oral screeners were prepared with disposable gloves, a disposable mouth mirror and a flashlight to check the athletes’ teeth. The examiners (*n* = 70) were dentists from university dental schools and dental professionals from private practices, who were previously trained and strictly calibrated according to the Training Manual for Standardized Oral Health Screening (supplement 1, supplement pictures 1a + b).

### Participants

**Fig. 1:**
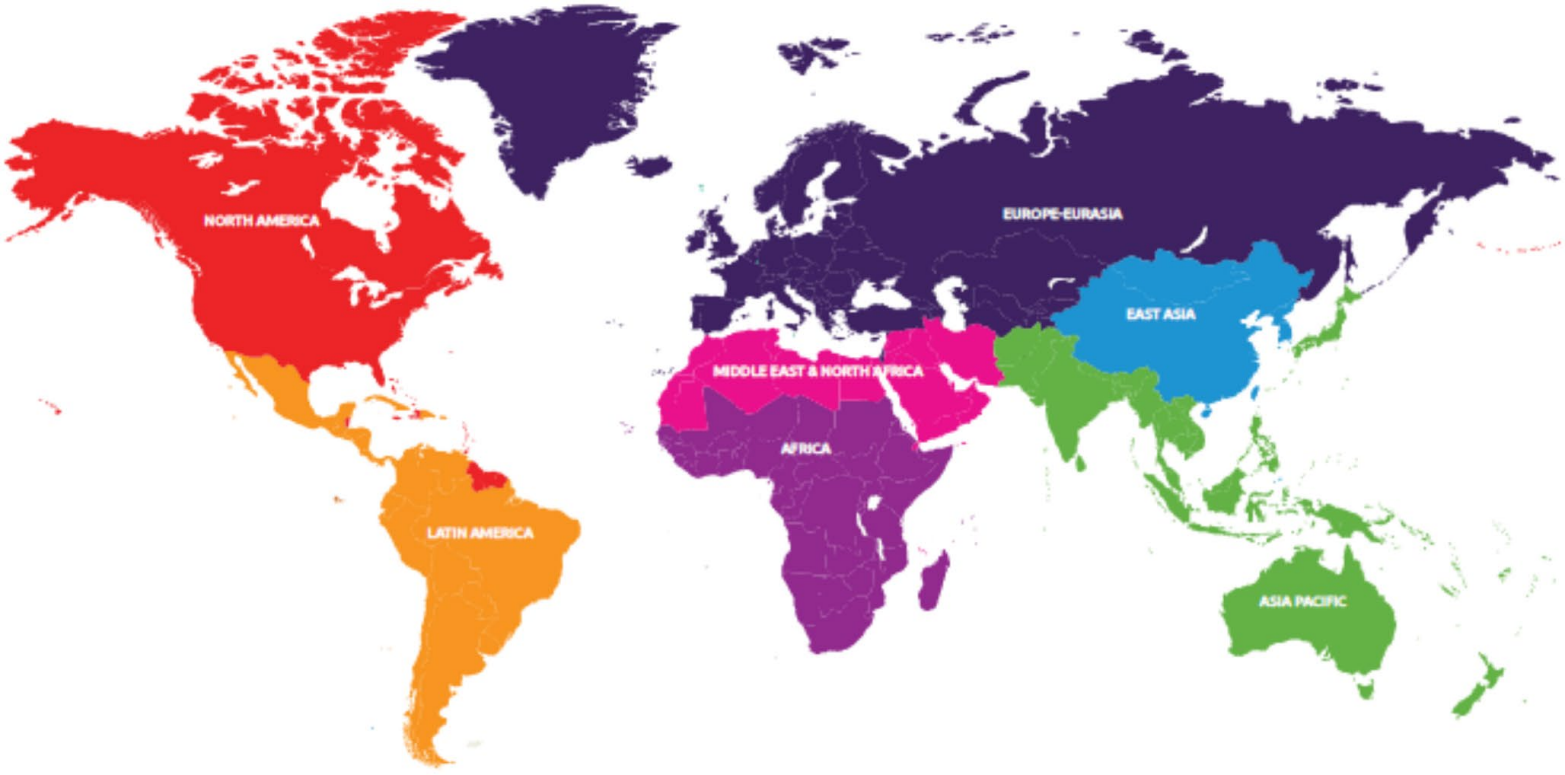
The seven different world regions according to classification of Special Olympics: Africa (light purple), Asia Pacific (green), East Asia (light blue), Europe/Eurasia (dark purple), Latin America (orange), Middle East North Africa (pink) and North America (red)

The target population were athletes with ID from all over the world participating at the Special Olympics World Games Berlin 2023. Special Olympics categorizes participants into the following world regions: Africa, Asia Pacific, East Asia, Europe/Eurasia, Latin America, Middle East North Africa and North America (Fig. 1). A sample size calculation was not performed, as the aim was to include as many participants as possible. The athletes were examined on a voluntary basis, while written consent from the athlete’s parent or guardian was obtained prior to the event. Athletes without signed consent did not participate in the SpecS program and no data was collected.

### Data sources/measurement

The standardized examination protocol, developed for SpecS by the US Centers for Disease Control and Prevention, Division of Oral Health, was strictly followed (supplement 1). This protocol involves the assessment of the oral cavity to determine the presence or absence of several conditions in separate cycles, independent of others. Third molars or partially erupted teeth were not included in this assessment.

The protocol is widely used and well accepted in literature [20, 22, 25].

### Variables

Records included the following demographic information: age, gender and date of birth, home country and region, sport discipline. Regions were classified according to the Special Olympics classification system. During the oral examination untreated caries, missing teeth, signs of dental trauma, gingival signs, fluorosis, fissure sealants, restorations and dental treatment needs were registered. The amount of missing teeth was classified as low if only anterior or only molars were missing, it was regarded as high if anterior and molar teeth were missing. According to the location of untreated lesions the amount of untreated lesions were classified as low if only anterior, premolar or molar teeth were affected, as moderate if a combination of 2 tooth types was affected and as high if all 3 types of tooth showed untreated lesions. With the aid of an interview, information on presence of pain, oral hygiene habits, and frequency of dental appointments were gathered and recorded.

Dental treatment needs were categorized into three levels of urgency based on the Special Smiles screening protocol (Supplement):

### Maintenance

No untreated decay, no untreated injuries, and no clinical signs of gingival inflammation.

### Non-urgent

No reported or observed oral pain, presence of broken fillings or gingival signs (e.g., bleeding), but no abscesses.

### Urgent

Presence of one or more of the following conditions: oral pain, possible pulpal involvement due to decay or injury, or presence of a periodontal abscess.

This classification was used to assess the need and urgency for follow-up dental care. In addition to the examination, all patients had an oral hygiene training and education session.

### Bias

In this study, several measures were implemented to minimize potential sources of bias. For the examination process, multiple examiners were involved, which introduces variability in assessment. To mitigate this risk, all examiners had received thorough training in standardized assessment and calibration procedures to ensure consistency in data collection prior to taking part in the oral examinations.

Regarding the questionnaires, responses were provided by athletes themselves using their unique IDs, which might have introduced bias based on individual comprehension and interpretation of the questions. To reduce this risk, companions were involved in the questionnaire process whenever feasible, and questions were formulated in “easy language” to enhance accessibility and improve the accuracy of responses.

### Study size

Data was collected as a convenience sample through interviews and oral examinations of all participating athletes from 17.06.2023 till 25.06.2023 who voluntarily presented to the Special Olympics World Games Berlin 2023.

### Statistical methods

Relative and absolute frequencies are given for categorical variables. Logistic and ordered logistic models were used to analyze univariate associations between the different target variables and the demographic variables region of origin, sport, age and gender. Finally, a multivariate analysis was carried out using the above models with the variables age, gender, region of origin and sport. All subsequent pairwise comparisons were corrected for multiple testing using Bonferroni.

In addition, the relationship between individual binary or categorical variables was examined using chi-square tests.

The significance level was set at 5%. All analyzes were performed using STATA 17.0 (College Station, TX, USA).

## Results

From about 7500 athletes with ID that participated during the Special Olympics World Games in Berlin, 2109 athletes participated in the oral health screenings (50.7% female, 49.3% male). The average age was 25.2 ± 8.7years. Participants were represented from seven different global regions (Fig. 1): Africa (13.8%), Asia Pacific (14.6%), East Asia (4.9%), Europe/Eurasia (30.3%), Latin America (13.9%), Middle East/North Africa (10.8%), North America (8.5%), and 3.2% with undeclared region.

The most common sports were athletics (22%), basketball (17%), bocce (9%), bowling (6%), and table tennis (6%) (Table 1). More detail is described in Suppl. Figure 2.

**Table 1:** Age, gender, oral health outcomes and region of origin of the athletes

Region	Number of participants (*n*)	Gender		Age (years)					Oral health outcomes (%)					
		Male (*n*)	Female (*n*)	Mean	SD	Min.	Max.	Median	Oral pain	Caries	Missing teeth	Dental trauma	Gingivitis	Fluorosis
Africa	291	130	161	22.69	6.9	15	57	21	15.5%	44.0%	26.5%	4.5%	34.0%	4.5%
Asia Pacific	307	158	165	22.85	7.3	15	57	21	9.1%	39.1%	20.8%	4.2%	34.9%	3.9%
East Asia	104	62	42	28.42	9.6	14	54	20	7.7%	39.4%	26.0%	3.8%	33.7%	6.7%
Europe/Eurasia	639	317	322	27.78	9.9	13	69	26	9.7%	37.2%	37.9%	3.9%	38.8%	1.3%
Latin America	294	132	162	25.39	8.7	12	59	23	13.6%	36.1%	35.4%	4.1%	37.4%	2.7%
Middle East/ North Africa	228	111	116	24.39	7.0	15	55	23	18.9%	55.3%	40.8%	5.3%	43.0%	3.9%
North America	179	92	71	26.43	8.8	15	58	24	17.9%	36.9%	29.6%	2.8%	26.3%	1.7%
Not declared	67	38	29	28.41	9.6	14	54	27	13.4%	46.3%	41.8%	6.0%	41.8%	4.5%
**Total (n/%)**	**2109/100%**	**1040/ 49.31%**	**1068/50.64%**	**25.23**	**8.7**	**12**	**69**	**23**	**267/12.7%**	**856/40.6%**	**688/32.6%**	**88/4.2%**	**772/36.6%**	**63/3.0%**

### Distribution of oral diseases

**Fig. 2:**
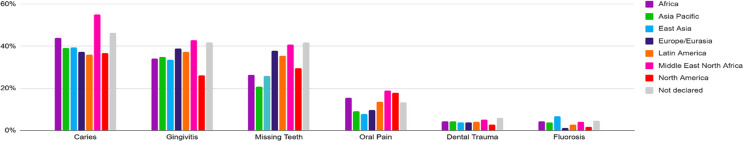
Prevalence of various oral diseases in athletes with intellectual disability according to the Special Olympics Regional classification (Fig. 1)

Oral pain was reported by 12.7% of the athletes (*n* = 267, female: 160, male: 107), with a regional variation from 7.7% in athletes from East Asia to 18.9% in those from middle east and North Africa (Fig. 2). Statistical analysis revealed a significant difference in the prevalence of pain amongst the regions (*p* = 0.001). The most severely affected region were Middle East/North Africa, North America and Africa. Those with less prevalence of self-reported oral pain were athletes from East Asia, Asia Pacific and Europe/Eurasia.

At least one caries lesion were present in 40.6% of the athletes (*n* = 856). Statistical analysis revealed a significant difference in the prevalence of untreated caries lesions amongst the regions (*p* = 0.001). No gender difference was shown (*p* = 0.2061). The highest prevalence was found in athletes from Middle East and North Africa (55.3%), while the lowest prevalence was in Latin America (36.1%) (Fig. 2; Table 1).

At least one missing tooth were observed in a total of 688 athletes (32.6% of those examined and having at least one missing tooth due to extraction), with a significant difference among regions (*p* = 0.001). Twice as many athletes from Middle East and North Africa had missing teeth when compared to those from Asia Pacific. There was no significant difference when comparing the type of sport practiced by the athletes and the prevalence of missing teeth (chi square *p* = 0.4339).

Dental trauma was present in 4.2% of the athletes (*n* = 88). Signs of dental trauma on the anterior teeth were not influenced by the type of sport the athletes conducted (*p* = 0.505) and also not influenced by the region of origin (*p* = 0.918).

Gingivitis was present in 36.6% (*n* = 772), with a significant (*p* = 0.006) regional variation from 43.0% in Middle East North Africa to 26.3% in North America.

Fluorosis was diagnosed in 2.9% of the athletes (*n* = 63). The regional prevalence varied, with a five-fold prevalence in East Asia (6.7%) when compared to Europe / Eurasia (1.3%) (Fig. 2; Table 1). Statistical analysis revealed a significant difference in the prevalence of fluorosis amongst the regions (*p* = 0.001).

### Oral hygiene habits

**Fig. 3:**
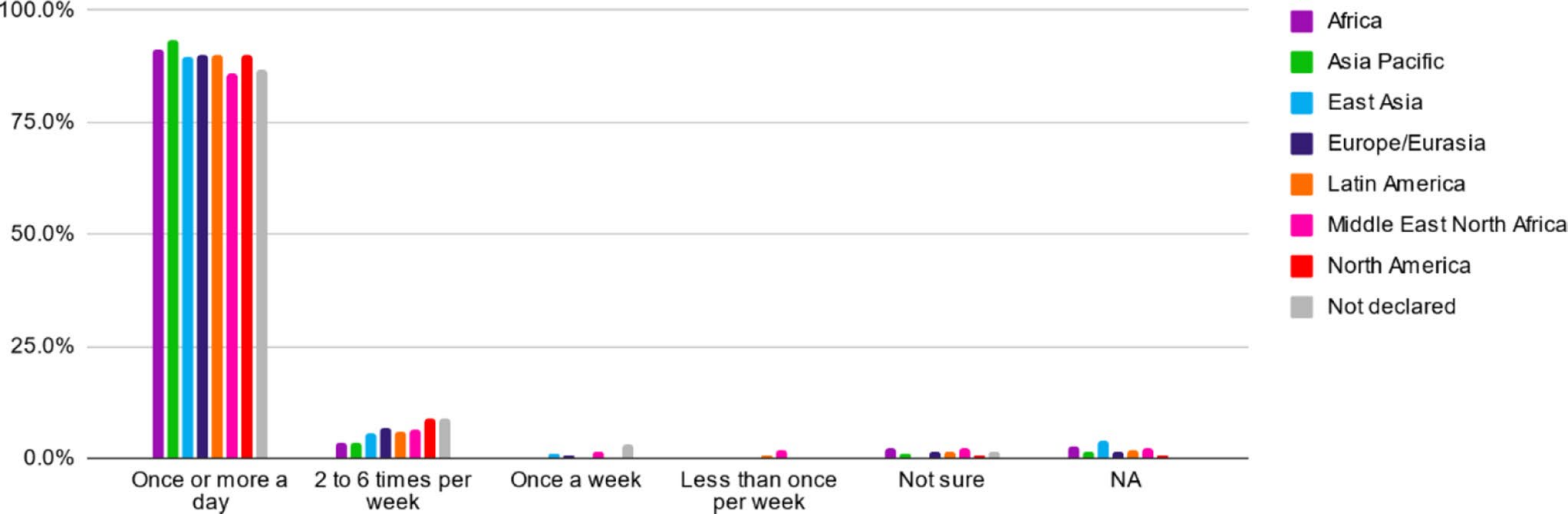
Reported frequency of oral hygiene practices among athletes with intellectual disability categorized according to the Special Olympics Regional classification (Fig. 1)

The data of the frequency of oral hygiene habits could be recorded in 98.3% (*n* = 2073) of the sample. The majority of athletes (90.1%) declared brushing their teeth at least once a day, while only 6% declared to brush 2 to 6 times per week (Table 2; Fig. 3). The lowest oral hygiene rate was observed in Middle East/North Africa with 87.9% and the highest in Asia Pacific with 95.3% brushing daily. A significant difference was observed between all regions (*p* = 0.038).

**Table 2:** Frequency of toothbrushing

Region	Total (*n*)	Toothbrushing (%)	
		At least once a day	less than once a day
Africa	283	93.3	6.0
Asia Pacific	318	95.3	4.7
East Asia	100	93.0	7.0
Europe/Eurasia	631	91.1	8.9
Latin America	289	91.7	8.3
Middle East/ North Africa	223	87.9	12.1
North America	162	89.5	10.5
Not declared	67	86.6	13.4
**Total**	**2073**	**91.70**	**8.3**

### Dental visits

The data of the frequency of dental visits could be recorded in 59.9% (*n* = 1263) of the sample. As athletes of the Special Olympics program are adults with intellectual disability, 40.1% of them were not able to respond to this question. Most athletes reported visiting the dentist once a year (32.3%) or even more frequently: twice a year (22.0%) and more than twice a year (12.4%). Only 11.7% scheduled their regular dental visits less than once a year, while another 21.6% only visited the dentist when they had dental pain. While no significant association in regard to age and gender was found, the frequency of dental visits differed significantly between the regions (*p* < 0.0001). In Middle East/North Africa, Africa and Asia Pacific about one third of the athletes visited the dentist only in case of dental pain. In contrast, in Latin America, East Asia and Europe/Eurasia more than 70% of the athletes visited the dentist at least once a year. Regional variability can be observed on Table 3.

**Table 3:** Dental attendance and region of origin of the athletes

Region	Total (*n*)	Dental attendance (%)				
		Only in case of toothache	< 1 a year	1 a year	2 a year	> 2 a year
Africa	105	32.38	12.38	33.33	18.10	3.81
Asia Pacific	155	30.32	9.68	32.90	18.06	9.03
East Asia	57	15.79	12.28	43.86	15.79	12.28
Europe/Eurasia	477	16.14	12.37	33.33	23.	14.68
Latin America	186	15.9	8.66	30.65	25.27	17.20
Middle East/ North Africa	127	34.65	8.66	33.86	13.39	9.45
North America	113	21.24	15.93	23.01	30.09	9.73
Not declared	43	20.93	9.30	27.91	27.91	13.95
**Total (%)**	**1263**	**21.62**	**11.72**	**32.30**	**22.01**	**12.35**

### Prevalence of fillings and fissure sealants

The presence of restorations was observed in 43.0% of the athletes, with a significant (*p* < 0.0001) variation among regions. The higher rates of fillings were present in athletes from Europe/Eurasia (58.95%) and lowest in those from Africa (11.11%). At least one tooth with a fissure sealing was only present in 10.4% of the cohort. Regional variation was tremendous, with higher frequency of sealants in Europe/Eurasia (14.8%) and only 1.5% in Africa (Table 4).

**Table 4:** Prevalence of restorative and preventive measures and region of origin

Region	Restorative/preventive measures (%)	
	Filled teeth	Fissure sealing
Africa	11.11	1.54
Asia Pacific	32.88	7.56
East Asia	48.42	12.90
Europe/Eurasia	58.95	14.80
Latin America	53.19	13.45
Middle East/ North Africa	43.08	8.29
North America	33.33	10.60
Not declared	42.19	7.94
**Total**	**43.02**	**10.39**

### Unmet dental needs

**Fig. 4:**
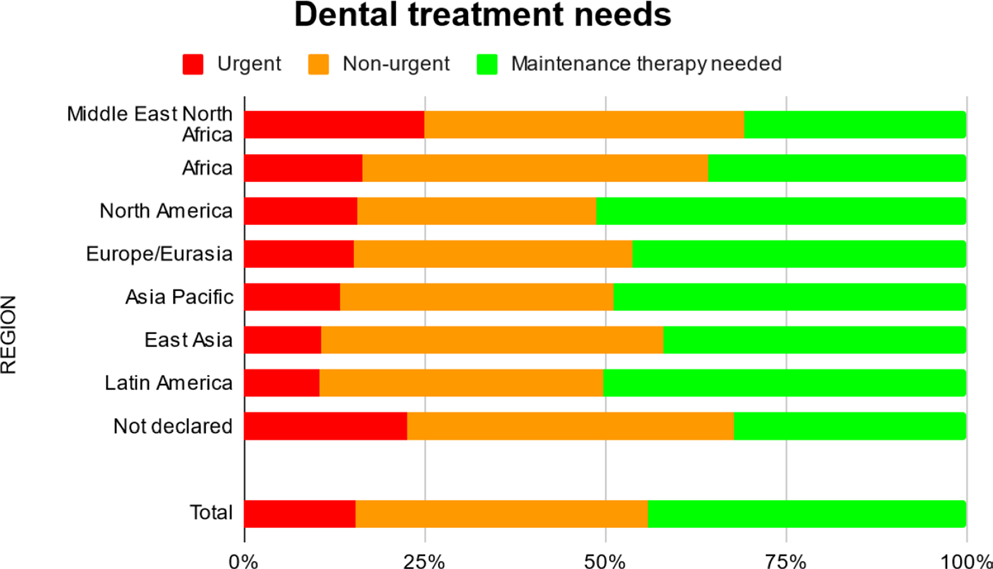
Dental treatment needs of special olympics athletes

Regardless of the region of origin, 55.9% of the athletes (*n* = 1061) exhibited a need for dental treatment. Moreover, 15.4% of the athletes required urgent dental care due to pain or infections. Significant differences (*p* < 0.001) between the regions were observed: Athletes from the Middle East and North Africa demonstrated the highest need for dental treatment (69.1%), while those from North America (48.8%) and Latin America (49.6%) exhibited the lowest need for dental care (Fig. 4).

### Univariate analysis of potential influencing factors on oral health outcomes

#### Age

Untreated caries (OR 1.04, p-value < 0.001), amount of missing teeth (OR 1.004, *p* < 0.001) and presence of gingivitis (OR1.02, *p* < 0.001) were higher with increasing age. While the diagnose of fluorosis was lower with increasing age (OR 0.949, p: 0.008), no influence of age was observed on caries experience (OR 0.999, p-value 0.886) and signs of dental traumata (OR 0.986, p-value 0.607).

#### Gender

Gender showed a significant influence on the level of untreated caries (OR 0.645, p: 0.004, in favor of females) and the occurrence of fluorosis (OR 1.89, p: 0.018, in favor for men).

#### Region of origin

The region of origin showed a significant association on the occurrence of pain (*p* = 0.001), fluorosis (*p* = 0.031), gingivitis (*p* = 0.006), level of untreated caries (*p* = 0.001), but no association to level of missing teeth (*p* = 0.535) and dental traumata (*p* = 0.920).

#### Sealants

A significant inverse association (*p* < 0.001) was observed between presence of fissure sealants and carious lesions. In the athletes with fissure sealants less untreated caries was diagnosed (OR 0.43) than in athletes without fissure sealants. Athletes with fissure sealants showed 52.9% prevalence of caries on molars, while those without sealants had a prevalence of 58.1%. The level of untreated caries for those athletes with fissure sealants was classified as low in 80.4% of them, and none (0%) had a high level of untreated caries. For the group without sealants, on the other hand, only 70% had low, 20% moderate and 10% had a high level of untreated caries.

#### Dental attendance

If the dentist was visited at least once a year neither the occurrence of carious lesions (OR 1.02; *p* = 0.868) nor the rate of lesions (low/moderate/high- OR 1.10, p-value 0.630) was significantly reduced. Dentists visit at least once a year showed no significant influence (*p* = 0.164/ *p* = 0.544/ 0.286) on a low/high amount of missing teeth (OR 0.65), gingivitis (OR 1.076) or dental traumata (OR1.79).

#### Frequency of tooth brushing

Daily tooth brushing significantly reduced the presence of carious lesions (OR 0.59, p-value 0.002) and the presence of missing teeth (OR 0.490, p-value 0.030). No significant influence was demonstrated for gingivitis (OR 0.903, p-value 0.543), level of untreated lesions (OR 0.80, p-value 0.341) or dental traumata (OR 1.83, p-value 0.557).

#### Multivariate analysis of potential influencing factors on oral health outcomes

If one examines the univariate results with a multivariate model, not all of the associations found hold. Age was significantly associated with the number of missing teeth (*p* < 0.001) and gingivitis (*p* = 0.006), while no significant influence was observed on oral pain, caries, dental trauma, or fluorosis. Gender showed a significant association with oral pain (*p* = 0.001), whereas no significant impact was observed on other parameters. Region demonstrated a strong association with oral pain (*p* = 0.003), untreated caries (*p* < 0.001), and missing teeth (*p* = 0.001), but had no significant effect on dental trauma, gingivitis, or fluorosis. The type of sport played by participants showed no influence with caries (*p* = 0.078) and dental trauma (*p* = 0.090) or other outcomes. Daily tooth brushing was significantly associated with a reduction in caries (*p* = 0.023), although no significant relationships were observed with other parameters. Finally, visiting the dentist at least once a year showed no significant associations with any of the evaluated oral health outcomes - Table 5.

**Table 5:** Multivariate analysis of health determinants and oral health outcomes (p-values)

	Oral Pain	Caries	Missing Teeth	Dental Trauma	Gingivitis	Fluorosis
Age	0.413	0.826	**< 0.001**	0.604	**0.006**	0.148
Gender	**0.001**	0.349	0.821	0.914	0.704	0.083
Region	**0.003**	**< 0.0001**	**0.001**	0.830	0.072	0.179
Kind of sports	0.588	0.078	0.738	0.090	0.879	0.506
Daily tooth brushing	0.704	**0.023**	0.082	0.852	0.312	0.649
Dental visits at least once a year	0.334	0.712	0.081	0.244	0.411	0.276

## Discussion

The Special Olympics Health Program is seen as a major facility in order to collect health data and making statements about the health of people with ID. With athletes from 152 countries participating, the results can be analyzed and compared in an international context. The results of this study provide important insights into the determinants of oral health in different population groups and their influencing factors. The analysis confirms the complex interaction between age, gender, region, preventive measures such as fissure sealants and the frequency of dental visits or oral hygiene practices. Sports and elite athletic performance can influence oral health, with ongoing discussions examining whether the stress associated with intensive training negatively impacts oral hygiene behaviors [26]. The athletes investigated in this study are individuals who, due to cognitive and in part also motor impairments, are often unable to independently maintain their oral hygiene, resulting in a particular need for support.

Analyses of the data regarding oral health in Special Olympics athletes with ID showed variability in the distribution of oral diseases between the seven Special Olympics regions. Region had a significant impact on several oral health parameters, including pain, fluorosis, missing teeth and untreated caries. This variability could be explained by differences in the availability and quality of dental care, water fluoridation, socioeconomic conditions and dietary habits between regions. The lack of association between region and dental trauma and missing teeth, on the other hand, could indicate more universal causes that occur independently of geographical factors. This is consistent with the outcome of previous studies [22]. People with ID are a heterogeneous group, also in terms of their ability to practice dental and oral care. In this study, athlete screening does not provide any information on the severity of the disability. It must therefore be assumed that differences in the frequency of dental and gingival disease are also due to differences in the quality of dental care. Studies show that the more severe the disability, the worse the oral health status [17].The support needed from carers for daily dental and oral care therefore needs to be individualized and adapted for people with ID.

Within the group of people with ID, Special Olympics athletes are likely to represent a privileged subgroup, characterized by higher levels of physical and mental fitness and health promotion in their living environment. Most participants in this study (30.3%) came from Europe/Eurasia, possibly because the event took place in Germany /Europe. It is likely that participation in the Special Olympics World Games is not possible for athletes with disabilities from very poor countries or rural areas. Due to the chance of a selection bias, the prevalence of oral diseases within this special group of patients may therefore be even higher than observed in this study.

Studies on oral health in people with ID conclude that those with ID have more missing teeth and fewer restored teeth than people of the same age without disabilities [27–30]. In our study, in athletes from MENA (40.8%) and Europe/Eurasia (37.9%) the highest proportion of persons with missing teeth was observed, suggesting that tooth extraction is often the preferred treatment among dentists in people with ID, rather than tooth preservation [31]. It is obvious that the average clinician is poorly trained or has limited experience and skills in dealing with this population of patients [32]. A lack of education in special care dentistry may have a negative impact on dentists’ attitudes and their willingness to treat patients with disabilities in their later professional lives [16]. This highlights the need for an early and comprehensive training in this field.

Most athletes (90.1%) reported brushing their teeth once a day. Daily tooth brushing showed a significant influence on the reduction of carious lesions but not on gingivitis. These results emphasizes the central role of oral hygiene together with the use of fluoride-based toothpastes in the prevention of caries but not in the prevention of gingivitis. As the athletes complete an oral hygiene program before the dental examination, it is possible that the information obtained influences their answer to this question. In addition, misreporting due to problems of understanding or lack of knowledge is possible. Limited dexterity, lack of understanding the toothbrushing process and the need for daily dental care are some of the main barriers for people with disabilities [33]. It can therefore be assumed that the reported frequency of tooth brushing does not necessarily reflect the quality of tooth brushing. Further studies should also ask whether dental care was done alone or with help, how long people brushed teeth, and whether they used an electric or manual toothbrush.

Caries prevalence is high in all regions. MENA (55.3%) and Africa (44.0%) top the respective list. As only visual inspection and no advanced methods of caries detection were used, the number of unreported teeth with caries lesions may be even higher. Regular sugar consumption is one of the main causes of tooth decay, and controlled sugar consumption significantly reduces the risk of tooth decay [34]. People with ID are at greater risk of developing tooth decay from sugar consumption, as they may also have poor health awareness and limited dental hygiene skills [35]. Sugar labelling on food and beverages and educational campaigns should be promoted globally to raise public awareness. To reach people with ID, information should also be available in accessible formats.

Urgent need for dental treatment following the Special Olympics criteria was observed in 15.4% of all athletes. With 55.9% presenting unmet dental treatment needs, participants from the MENA region were particularly affected. A third of the participants from these countries (34.7%) reported that they visit the dentist only in case of pain. These alarming facts once again point to a major imbalance in global healthcare systems and make international efforts to address them essential.

Fissure sealing (FS) was detected in only 10.4% of all participants, although our analyses showed that this prophylactic measure contributes to a significant reduction in carious lesions. FS has been described in the literature as an effective way of preventing tooth decay in children and adolescents [36]. However, people with disabilities are much less likely to receive FS [37, 38]. As FS is a non-invasive and short-term procedure that does not require anaesthesia or drilling, it can work well for patients even with limited cooperation. FS should therefore be provided at an early stage in people with ID and should be carried out as a routine measure.

The risk of untreated caries, gingivitis and tooth loss increases with age in people with ID [28], which is consistent with the present findings. It can be assumed that the younger athletes are more likely to live with their families and receive dental care support from them. Close supervision of oral hygiene is therefore needed at all stages of life to avoid the negative effects of tooth decay on speech, nutrition, general health and quality of life in old age [30, 39].

A systematic review of oral health outcomes from people participating in the Special Olympics over the last two decades shows that the oral health status of people with ID has not improved within the years [23], suggesting that their oral health is worse compared to the general population [40, 41]. Measuring the exact effectiveness of oral health promotion and programs is challenging unless they are consistent and sustained over time. Educational interventions focused on oral health promotion have shown benefits for individuals with IDs and their caregivers by improving knowledge, attitudes, and oral health behaviors. However, these interventions need to be implemented over the long term to ensure lasting impact [42].

### Limitations

Although the protocol used in the present study is widely accepted in literature [20, 22, 25, 43, 44], the study has some clear limitations that need to be considered when interpreting the results. The findings of this study should be interpreted with caution, as they cannot be generalized to the entire population of individuals with ID. The participants represent a specific subgroup of athletes in the Special Olympics, who may receive higher levels of support but are not necessarily less dependent. Additionally, the convenience sampling method and varying sample sizes across countries introduce potential selection bias.

Participants in this study are likely to have better physical health and cognitive abilities than the broader ID population, given that the coordination and rule-following skills required for regional events may skew the sample toward a healthier subset. Self-reported data on oral hygiene habits, visit to the dentist and pain may also be subject to bias, as athletes might provide responses that they perceive as socially desirable, potentially leading to overestimations in reported oral care behaviors.

Although all examiners were trained using standardized protocols, no formal statistical calibration was conducted. This may have led to variability in the application of criteria and examination procedures, despite consistent training. A further limitation of this study is the lack of intra- and inter-observer reliability assessment during the validation process, which may affect the consistency and reproducibility of the recorded findings.

Finally, addressing oral health disparities in individuals with ID requires tailored, community-specific strategies, highlighting the need for ongoing efforts from policymakers, healthcare professionals, and caregivers to reduce these disparities and mitigate the vulnerabilities of this population.

## Conclusion

This study underscores significant global disparities in the oral health of athletes with ID and reveals notable regional variability in access to preventive and restorative dental care. Although many athletes report regular dental visits and daily brushing, the high prevalence of untreated caries, gingivitis, and missing teeth highlights persistent unmet dental needs in this population. The findings emphasize the necessity of specialized dental care tailored to the unique needs of individuals with ID, including behavioral interventions, caregiver education, and preventive measures such as fissure sealants and fluoridate toothpaste.

## Electronic supplementary material

Below is the link to the electronic supplementary material.

Supplementary Material 1

Supplementary Material 2

## Data Availability

No datasets were generated or analysed during the current study.
