# Supplementary material for: Oral health of individuals with intellectual disabilities: a global cross-sectional study from the special olympics world games 2023
Source: Clin Oral Investig. 2025 Apr 16;29(5):252. doi: 10.1007/s00784-025-06331-3 (PMC12003447; doi:10.1007/s00784-025-06331-3)
Supplement: Supplementary file 1 — Supplementary Material 1 [file 784_2025_6331_MOESM1_ESM.pdf]

Training Manual for Standardized Oral Health Screening

Revised Edition November 2004

Division of Oral Health
National Center for Chronic Disease and Health Promotion
Center for Disease Control and Prevention

Judy A. White, R.D.H., M.P.H.
Eugenio D. Beltran, D.M.D., Dr. P.H.
Steven Perlman, D.D.S., M.ScD.

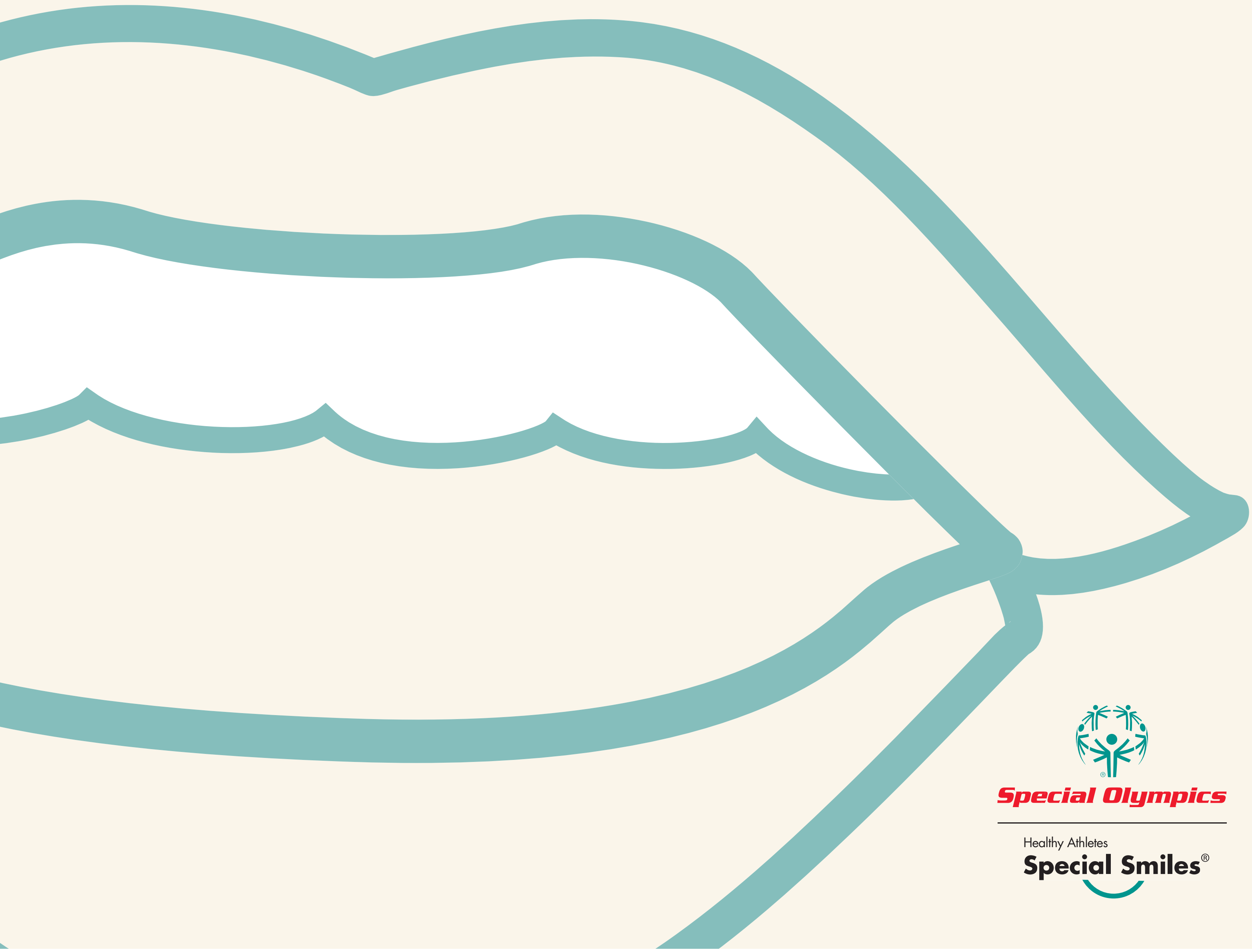

Special Olympics

Healthy Athletes
Special Smiles®

Training Manual for Standardized Oral Health Screening

Division of Oral Health
National Center for Chronic Disease and Health Promotion
Centers for Disease Control and Prevention

Judy A. White, R.D.H., M.P.H.
Eugenio D. Beltran, D.M.D., M.S., Dr.P.H.
Steven Perlman, D.D.S., M.ScD.

Revised Edition
November 2004

© Special Olympics, Inc. 2004
All rights reserved.

This manual was originally produced by the Centers for Disease Control and Prevention, National Center for Chronic Disease Prevention and Health Promotion, Division of Oral Health. It was prepared by the Division of Oral Health to meet the needs of the Special Olympics Special Smiles® dental health program.

We thank staff members of the Division of Oral Health who made recommendations and reviewed earlier drafts, particularly Dolores M. Malvitz, Dr.P.H., and Stuart A. Lockwood, D.M.D., M.P.H.

We thank Special Smiles Clinical Director Steven Perlman, D.D.S., M.ScD., the Special Smiles management team, and all of the associated logistical and advisory professionals who offered comments along the way.

We thank those who contributed examples of earlier screening materials for the first draft of this manual from Special Smiles sites in Georgia, Maryland, Massachusetts, New Jersey and Pennsylvania. We also thank the site coordinators, dental screeners, recorders and other volunteers in the three pilot sites of Boston, Newark and the San Francisco Bay Area for participating in and contributing feedback about the standardization process in the first pilot year.

We thank the talented staff of the Centers for Disease Control and Prevention, whose support was essential to the completion of this project: Carol Hudlow and Darian White.

Suggested Citation

White *JA* and Beltran ED. *Training manual for standardized oral health screening.*

Atlanta, GA: Centers for Disease Control and Prevention, National Center for Chronic Disease Prevention and Health Promotion, 1998

© Special Olympics, Inc. 2004
All rights reserved.

Dear Dental Screener:

Since the inception of Special Olympics Special Smiles, one of the primary goals of the program has been to collect standardized data on the oral health of Special Olympics athletes. The format that we use was developed by a collaborative effort between Special Smiles and the Division of Oral Health at the Centers for Disease Control and Prevention (CDC). In 1997, this protocol was tested at three events. The standardized data that was collected was reported and provided information on the oral health status of Special Olympics athletes in their city. It was demonstrated that the program could become a most important vehicle to collect data on this hidden population.

As the program has significantly expanded, tens of thousands of athletes have been screened and accessed into the health care system where they have received treatment that may not have been available to them previously.

Special Smiles is now a component of the Special Olympics Healthy Athletes® initiative. Founded in 1996, this program is designed to help athletes improve their health and fitness and improve their ability to train and compete in Special Olympics as well as life.

The program has been directly responsible for several historic milestones that have led to improvements in health care for children and adults with intellectual disabilities. In September 2000, Healthy Athletes collaborated with Yale University to publish "The Health Status and Needs of Individuals with Mental Retardation."

By using this document to create awareness and advocating its importance, Healthy Athletes led the United States Senate to hold its first hearing on this issue. This historic event was held at the Special Olympics World Winter Games in Anchorage, Alaska (USA) on 5 March 2001. At the hearing, U.S. Surgeon General David Satcher agreed to hold the first-ever Surgeon General's Conference on "Health Disparities and Mental Retardation" which came to fruition on 5-6 December 2001 in Washington, D.C. As a result of the conference, a report was released entitled "Closing the Gap: A National Blueprint to Improve the Health of Persons with Mental Retardation."

The Healthy Athletes program's credibility and expertise in data collection was also responsible for its participation and testimony in U.S. Senator Bingamen's Children's Oral Health Hearing on 25 June 2002.

With this extraordinary success, it is our goal to continually expand standardized data collection sites to enable us to report region-specific information that may be used by advocacy groups to improve access to dental care for people with special needs. Your willingness to participate in our program is essential to this effort. This manual will provide you with the necessary information to become a standardized screener and "public health" dental professional.

Thanks again for your participation. We look forward to working with you and seeing you at a Special Olympics event.

Steven Perlman, D.D.S., M.Sc.D.
Global Clinical Advisor
Special Olympics Special Smiles

© Special Olympics, Inc. 2004
All rights reserved.

TABLE OF CONTENTS

Why Standardize? **1**

Healthy Athletes Software (HAS) System Form **2**

Guidelines for Data Coordinators **3**
- Introduction 3
- Planning for the Event 4
  - Recruitment of Volunteers 4
  - Training 4
  - Registration 6
    - - Screeners 6
    - - Recorders 6
  - Checklist of Materials Needed 6
  - Other Planning Considerations 7
- Day of the Event 8
  - Registration 8
  - Training 8
  - The Screening Process 8
- After the Event 9

Screeners **10**
- Objectives for Screeners 10
- Case Definitions and Instructions 10
  - Event Specifics 11
  - Dental History 12
  - Screening 12
    - Edentulous 12
    - Untreated decay 13
    - Filled teeth 15
    - - Special Considerations Associated with Anterior Teeth 16
    - Missing teeth 16
    - - Eruption Dates for Use in Determining Missing Teeth 18
    - Sealants 19
    - Injury 19
    - Fluorosis 21
    - - The Differential Diagnosis of Fluoride and Non-Fluoride Enamel Opacities 23
    - Gingival signs 24
    - Treatment Urgency 25
    - Mouth guard recommended 26
    - Exam complete 26
- Procedures for Screeners 27
- Screening Procedures Summary Chart 30

Recorders **31**
- Objectives for Recorders 31
- Screening Procedure for Recorders 32
- Tips for Recorders Using Screening Forms 34

© Special Olympics, Inc. 2004
All rights reserved.

TABLE OF CONTENTS

Practice Test **35**
- Standardization Exercises 35
- Standardization Exercise Photos 38
- Standardization Exercise Answers 39

Non-Clinical List of Instructions for Training Sessions **40**

Data Cover Sheet **41**

Contact Information **42**

© Special Olympics, Inc. 2004
All rights reserved.

Why Standardize?

While an oral screening of a group of people parallels the methods used in clinical practice on individuals, it also differs from those methods in several important ways. A **diagnostic oral examination** in clinical practice is a compilation of signs, symptoms, diagnostic test results and professional judgment that identifies the needs of a **person**, from which a treatment plan can then be developed. An **oral screening** is a compilation of visual evidence of the pathology present in groups of people that helps identify the needs of a **population**, from which their “treatment” (services) can then be planned.

Measurement of oral health status and changes in that status over time necessitates the screening of samples of the population, and more than one screener usually participates. Due to inherent potential variability among screeners, the criteria used to measure oral conditions in the **population** must emphasize reproducibility of results rather than meticulous detection of the earliest signs of disease, as in the case of individual **persons**. The process by which these criteria and methods are internalized by screeners and their recorders is called **standardization**.

Standardization is very important if data are to be collected. Standardization of the screeners on the basis of defined criteria reduces the human nature of bias (which exists in part as a result of clinical education and experience), and it is the means by which we can help ensure that the results of the oral screening are valid (correctly categorizes persons into disease/no disease categories) and reliable (criteria have been applied consistently). Standardization allows data from several sites to be combined.

This standardization of all screeners in the consistent application of diagnostic criteria increases **inter-examiner reliability**, which can be increased by studying this document beforehand and participating in the standardization training on the day of the event. **Intra-examiner reliability** (the ability of one screener to consistently call the same findings) is not addressed at this time because athletes have only a few minutes between events to go through the screening, and asking athletes to participate in the screening once is already a significant contribution on the one day a year this event takes place.

In order to meet the goal of providing an accurate, consistent assessment of the oral conditions observed, it is important that, for this event, you set aside your own professional judgment on whether, for instance, a tooth examined would represent decay in a clinical setting. In this oral screening, you would mark it *only if it has decay according to the case definition established*. Please remember that the ability to screen in a standardized way is not a measure of your clinical skill. Rather, by screening in this way, you will help in the accurate assessment of this population while *still* providing a valuable referral to the athlete for oral conditions that need follow-up.

In this manual you will find the case definitions for the oral screening at Special Olympics’ events. It is important to review them before the day of the event if possible. Thank you for your interest, time and effort in participating in the Special Olympics oral health screening and for your cooperation in helping Special Olympics Special Smiles collect consistent data.

© Special Olympics, Inc. 2004
All rights reserved.

Healthy Athletes Software (HAS) System Form: Special Smiles

All the Healthy Athletes disciplines use a standardized form (see Special Smiles form below) to collect data during athlete screenings. Through a Web-based software application called Healthy Athletes Software system (HAS), screening data is captured electronically, allowing Healthy Athletes to compile a large, high-quality health database on individuals with intellectual disabilities. Athlete data can be captured on paper, then entered into the HAS Web site at a later time. When the HAS system development is complete, the ideal situation will be to have Web access and available PCs (personal computers) at the screening event. Another option will be the use of wireless PDAs which can transfer the data directly to the Web site or via a PC. For now, please forward the paper HAS forms to Special Olympics headquarters where the data will be entered into the system.

Firstname	Lastname	HASS ID ____ ____ ____ ____ ____

Date	O Male O Female	DoB	Age (years) O Not sure
World Games 2003	Dublin, Ireland	O Athlete O Unified partner	Sport

Delegation	Region	Country

Screener's name

Dental History

1. Fill out this section for each athlete even if edentulous

How often do you clean your mouth?

O Once or more a day

O 2 to 6 times per week

O Once per week

O Less than once per week

O Not sure

2. Pain inside mouth

O Yes O No

☐ Teeth

☐ Other

3. ☐ **Athlete refused/could not screen**

Screening

4. Edentulous

O Yes **(-> stop here)** ☐ **Exam completed**

O No **(answer all questions 5 thru 14)**

5. Untreated decay

O Yes O No

☐ Anterior(s)

☐ Premolar(s)

☐ Molar(s)

6. Filled teeth

O Yes O No

7. Missing teeth

O Yes O No

☐ Anterior(s)

☐ Molar(s)

8. Sealant(s)

O Yes O No

9. Injury

O Yes O No

10. Fluorosis

O Yes O No

11. Gingival signs

O Yes O No

12. Treatment urgency

O Maintenance

O Non-urgent

O Urgent

13. Mouthguard recommended

O Yes O No

☐ Mouthguard delivered

14. ☐ **Exam completed**

Special Smiles Page 1 of 1

Special
Smiles

© Special Olympics, Inc. 2004
All rights reserved.

Guidelines for Data Coordinators: Initiating Standardization at Your Special Olympics Special Smiles Site

Introduction

Thank you for your time and effort in coordinating standardization of data collection at your Special Olympics Special Smiles oral health screening site!

As Data Coordinator, you have four main goals:

- To train the dental screeners to enable them not only to understand the case definitions, but to apply them consistently;
- To train dental recorders to record the information in a way that it will be usable in data analysis and reporting;
- To be available throughout the actual screening process to ensure that every screener has gone through the standardization training, and to answer questions as they arise (and they will); and
- To ensure that a Screening Form has been collected on each and every athlete who participated in the screening and that the resultant data are forwarded appropriately for analysis.

Your willingness and diligence in managing the quality assurance of this process are essential to the success of standardized data collection, which allows data to be compared or combined with data from other sites. Specific standardization data on this population can be valuable to your own state/province as well as in learning more about this population and directing advocacy efforts or services.

Work closely with your Special Smiles Site Coordinator from the beginning to avoid duplication of effort in common areas of activity, such as scheduling of screeners and recorders, issuance of badges and screening area set-up. Exchange and discuss guidelines and manuals (e.g., for site and data coordinators) early so you are aware of potential areas of overlap as well as areas that you alone will be responsible for.

These guidelines are designed to help you achieve and enjoy a smooth standardization process.

© Special Olympics, Inc. 2004
All rights reserved.

Planning for the Event

Recruitment of Volunteers

Your Special Smiles Site Coordinator may appreciate help in recruiting volunteer screeners and recorders. Dental and hygiene schools, and their associations and local societies are obvious sources for screeners. Depending on your state or province's practice act, dental hygienists may be able to screen as well as provide oral hygiene education.

Your site may have a master list of screener and recorder names, addresses and telephone numbers from the previous year. If your site does not have a master list, be sure to start one this year; you will need it to schedule standardization training sessions, to send case definitions in advance of the screening date and to develop a listing for future years.

As Screeners are calibrated and standardized:

- Explain that the event includes a standardization training session (you may want to refer to page 1, "Why Standardize?" to answer any questions). It is important to explain that, while contribution of professional expertise on event day is acknowledged and appreciated, cooperation through punctuality in attending a standardization training session in its entirety allows the benefits of the screener's contribution of time to extend far beyond the day of the event. To ensure standardized data, everyone must complete standardization training prior to their screening.
- Confirm the training session the screener will be attending. Work with the Special Smiles Site Coordinator to identify times of the standardization training and the continuing education (CE) course.
- Get a telephone number, mailing address and e-mail address from your volunteers in case you are able to send the case definitions in advance of the screening date. Screeners have reported that it is helpful to review the case definitions before the standardization training session. Providing them with this opportunity is highly recommended if possible, even if the screeners receive the information only a day or two before the event. An abbreviated version of the case definitions is included for early distribution and review. Important: Do not distribute or use the abbreviated version for the standardization training session and/or on the day of the event; use only the full manuals at that time. For adequate standardization, the screeners must have use of the full manual (which includes color photos and attachments) for training and screening.

Sometimes unanticipated shortages of a particular type of volunteer occur on the day of the event. For example, your site may find itself suddenly short of several recorders, oral hygiene instructors or registrars, creating bottlenecks at stations before or after the screening process. You may want to mention to the screeners how much it helps the whole effort if they arrive willing to be flexible and to handle other positions during their shift, even temporarily.

Training

The shortest total time for each standardization training session (not including the continuing education course for Special Smiles) is approximately 45 minutes: 30 minutes for the presentation plus 15 minutes for the standardization exercise and the question-and-answer period. It is unlikely that the concepts necessary for the uniform application of the case definitions could be conveyed or absorbed in a shorter period of time to the point where they could be consistently applied, thus jeopardizing the "standardized" element of the data.

© Special Olympics, Inc. 2004
All rights reserved.

The suggested methods are based on feedback from screeners from many sites regarding strategies that were most useful in applying case definitions to the screening process. Every screener should attend a training session that includes the following:

- A presentation in which the Site Data Coordinator goes through the case definitions and photos in the manual;
- A standardized exercise; and
- A question-and-answer period in which the standardization exercise is discussed.

A brief, solitary review of the manual by screeners upon arrival allowed room for individual rather than standardized interpretations when applying the case definitions. Also, screeners who arrived late and observed a standardized screener as their only training before screening were not able to consistently apply the subtleties of the case definitions.

It has been found helpful to schedule the standardization training before the continuing education (CE) course; this allows screeners who have taken the CE course in previous years to begin screening. All screeners should attend the standardization training each year, whether or not they have attended standardization training previously.

It is advisable to plan for two standardization training sessions: one for the morning volunteers before the screening begins and one for the afternoon shift around midday. The session scheduled before the screening begins could be given on the evening before the event or on that morning. Be sure to schedule the standardization training times to allow the afternoon shift to arrive and complete the training before the morning shift leaves. Be prepared to offer an impromptu (but complete) standardization training session or two as late screeners arrive; signing up screeners beforehand (rather than letting screeners make up their own schedules) will reduce the need for impromptu training. The Site Data Coordinator and the Site Coordinator, as well as yourself, can offer the option of other volunteer activities for screeners who cannot commit to a morning or afternoon standardization training session, or arrive too late to attend.

Experience has shown that it is very important to conduct the standardization training in a quiet area, away from all the activity, so that screeners will not be distracted and displaced as the venue begins to fill with athletes and volunteers. You will not need electricity or chalkboards for your presentation; therefore, the training area can be anywhere from a semi-secluded spot within the Special Olympics Village (although rain could be a factor in this case) to an air-conditioned classroom reserved within walking distance. The important thing is to identify the spot beforehand, and use it the entire day, so latecomers can be directed to you.

Be sure to have enough manuals to train the volunteers (two screeners could share a manual) and a copy of the standardization exercise (included) for each screener (screeners should not fill out the exercise in the manuals, as manuals will be used from year to year). Contact your Special Smiles person in charge of details at the sites. The manuals should not go home with the screeners, but remain at the site.

Screeners have found it helpful to have a reproduction of the Screening Procedure Summary Chart in the manual readily available for quick reference in the screening area. Check with Special Smiles for availability of this chart in poster form for display in the screening area, or provide copies at each screener/recorder station (perhaps in a sheet protector) for quick reference.

© Special Olympics, Inc. 2004
All rights reserved.

GUIDELINES FOR
DATA COORDINATORS

Before your event, you will be trained on how to train the screeners in your area. Details of these train-the-trainer sessions are available through Special Smiles.

Registration

Screeners:
As mentioned, inconsistent standardization can result in unreliable data that cannot be compared or combined with data from other sites, or even used by itself. Because of the importance of ensuring that every screener has gone through standardization training, a plan of action for any contingency that may arise should be developed. For example, have a plan of action if screeners arrive after the training session has progressed past the introduction and assignment of ID numbers; after the training session is finished; or join the screening process without being trained.

Recorders:
While not as involved as the training for screeners, training for all recorders is mandatory to avoid mistakes (which necessitate entry of the HAS Form by hand) and ensure completeness of collected data. The recorders can be scheduled as a group or individually; allow about 15 minutes before recorders are scheduled to start working.

All recorders should be given the following sections of this manual as a complete packet:

- Objectives for Recorders
- Screening Form
- Screening Procedures for Recorders
- Tips for Recorders Using Screening Forms or Tips for Recorders Using Direct Data Entry (depending on the type of recording system at your site)

While these sections can be found in the training manual, the availability of separate copies will allow screeners to use manuals at the same time recorders are reading the instructions. A copy of the materials for recorders is attached.

Checklist of Materials Needed *(review with local Special Olympics Healthy Athletes contact)*

- Flashlights with extra batteries
- Tongue Blades
- Toothpicks (for checking for sealants; see procedure sequence for dental screeners)
- Gloves (latex/non-latex)
- Name Badges
- Pencils with erasers
- Boxes or bins for completed forms (enough to place at each or between screener/recorder stations)
- Screening Forms
- Report Cards

© Special Olympics, Inc. 2004
All rights reserved.

- Enough manuals for each screener or pair of screeners
- Copy of standardization exercise for each screener
- Posters or copies of the Screening Procedure Summary Chart
- Instructions for Recorders (compiled as described)
- Miscellaneous items (scissors, strong tape, pushpins, yarn/string, pencil sharpener, hole punch, markers)

Other Planning Considerations
Plan with the Site Coordinator on the following:

- Establish the sequence of stations (e.g., is the screening held before or after oral hygiene education) and where the athlete will be directed after completion of the screening. Instruct the screeners and recorders accordingly.
- Decide who will fill out the Event Specifics and Athlete Demographics at your site (e.g., registrars or recorders); include this information in the instructions for the screeners and recorders. The screener and recorder always fill out the Examiner ID category and the Dental History and Screening Findings section.
- Determine the appropriate method for the disposal of gloves, tongue blades and toothpicks.

© Special Olympics, Inc. 2004
All rights reserved.

Day of the Event

Registration

As Data Coordinator, you will be in charge of registering the screeners and recorders as they arrive. Name badges will be issued to the screeners at the time of registration; you and the Site Coordinator should agree beforehand on who (e.g., you or the registrars) will issue the badge to the screeners and recorders.

Keep track of how many screeners were trained. Be sure that all screeners and recorders sign in on a master list, and include their telephone numbers and addresses.

Manuals should be available at the screening tables for each screener/recorder team to use as a reference throughout the day.

Be sure that all volunteers know the type of event, and any criteria for filling out additional information (site use) on the Screening Form.

Training

You will assign a unique three-digit Examiner ID number (starting with 001) to each screener as he/she arrives for the standardization training. Writing this number on the badge is invaluable in helping the screener remember his/her number and in identifying which screeners have or have not completed the standardization training.

For your presentation, you may want to offer some introductory remarks on why Special Smiles wants to collect data, and how it could be useful locally. Explain briefly why and how data is standardized (refer to the attached sheet, “Why Standardize?”). The key point to emphasize is the reason for the necessity of setting aside professional clinical judgment to examine and diagnose individuals, and screen according to the case definitions to “diagnose” a population. Then go through the Case Definitions and Instructions Section in the manual.

After your presentation, pass out the standardization exercise and allow the screeners a few minutes to complete it individually; do not complete it as a group. The photos referred to in the exercise are in the back of the manual; instruct the screener to fill out the distributed copy of the standardization exercise rather than the exhibit in the manual. The exercise will be a starting point for discussion as you go through the rationale for the answers. The exercises and the questionnaire on the last page continue to be used to evaluate the training process; collect the exercises and send them to the specified address.

The Screening Process

Remain near the screening area to answer any questions and to train any screeners who arrive after the training session.

Screeners may be asked to screen coaches coming through with their teams. This is a courtesy screening only; do not fill out a Screening Form for anyone other than athletes, as they are the only group for whom data are being collected at this time.

Enjoy the event!

© Special Olympics, Inc. 2004
All rights reserved.

After the Event

Important: Please send the HAS Forms or disks and the completed Standardization Exercises immediately to the location specified by Special Smiles for analysis.

Store or return the manuals and other standardization material as directed by Special Smiles.

Thank you again for your time and effort in coordinating standardization of data.

© Special Olympics, Inc. 2004
All rights reserved.

Special Olympics

Healthy Athletes Special Smiles®

Objectives for Screeners

At the completion of training, the screener will be able to:

1. Recognize the difference between a diagnostic oral examination in clinical practice and an oral health screening of a sample of a population;
2. Understand the importance of standardization in a screening process;
3. Identify the conditions that are being recorded in this screening;
4. Know the case definitions for the conditions being recorded; and
5. Consistently apply the case definitions to the screening process.

Note: The following pages provide a step-by-step guide to filling out the Special Smiles Healthy Athletes Software system (HAS) form.

Case Definitions and Instructions

Event Specifics

- Record the first name, last name and Healthy Athletes Software system (HAS) ID number if available.

First Name	Last Name	HASS ID ___ ___ ___ ___ ___

- Record the date of the event in the box provided.
- Sex – Record the appropriate answer: male or female
- Record the date of birth (DoB) if available. Ask the athlete: "How old are you?" Record the response. If the athlete does not know, mark "Not sure."
- Write the name of the event and the location in the appropriate box.
- Check the appropriate circle: Athlete or Unified Partner
- Record the athlete's Delegation, Region and Country if appropriate.

Date	○ Male ○ Female	DoB	Age (years) ○ Not sure
Event:	Location	○ Athlete ○ Unified Partner	Sport
Delegation	Region	Country	

- Write the screener's name in the appropriate box.

Screener's name

© Special Olympics, Inc. 2004
All rights reserved.

Dental History (Sections 1-3 on the screening form)

Fill out this section for each athlete, even if edentulous.

1. The question, “How often do you clean your mouth?” is used rather than “How often do you brush your teeth?” because we want to record *frequency* of oral hygiene *effort* regardless of the specific (adaptive) devices used.

Ask the athlete “How often do you clean your mouth?” and mark the phrase that best fits his/her response. If the athlete isn’t sure how often he/she cleans the mouth or does not understand, mark “Not sure.”

Dental History

1. Fill out this section for each athlete even if edentulous

How often do you clean your mouth?

- O Once or more a day
- O 2 to 6 times per week
- O Once per week
- O Less than once per week
- O Not sure

2. Pain inside mouth:

Ask the question “Do you have any pain inside your mouth now?”

If the athlete answers “**Yes**,” mark “**Yes**” and then say, “Point to the place where it hurts.”
Mark “**Teeth**” if athlete points to teeth.
Mark “**Other**” if athlete points to palate, soft tissue, gingival or any other area in the oral cavity.

If the athlete answers “**No**,” mark “**No**.”

2. Pain inside mouth

O Yes O No

- ☐ Teeth
- ☐ Other

3. Ask the athlete “Can I look at your teeth today?” If yes, continue. If no, mark “Athlete refused/could not screen” on the form and send the athlete to the next station. Keep the screening form.

□ Athlete refused/could not screen

© Special Olympics, Inc. 2004
All rights reserved.

Special Olympics

Healthy Athletes Special Smiles®

Screening (Sections 4-14 on screening form)

Important: Please apply the following guidelines to the clinical portion of the screening.

a. This screening is person-based. The *number of persons* with or without a condition is being assessed rather than the *number of surfaces* or the *number of teeth* with or without a condition.

b. Assess each condition in a *separate* cycle, independent of all other conditions. A cycle is one visual tour of the mouth, starting over from the same point (e.g., top right to left, then bottom left to right) or from the point specified for each condition. For example, during the visual cycle for untreated decay, do not look for untreated decay *and* fillings, look for untreated decay only. This reduces the chance for errors and reduces confusion for the recorder.

c. If two conditions are present on the same tooth, mark both. For example, mark a filled tooth with recurrent decay under both the "Untreated decay" and "Filled teeth" categories.

d. Consider all visible surfaces on orthodontically banded teeth.

e. For this screening, *do not* consider the following:
- third molars
- partially erupted teeth (teeth that have not reached occlusion)

4. Edentulous

Mark "Yes" if:
- no teeth are present, **and stop here**. Mark "Exam completed" on the form and direct the athlete to the next station.

Mark "No" if:
- one or more teeth are present; or
- root tips only are present.

Continue with the screening.

Screening

4. Edentulous

O Yes (-> stop here) ☐ Exam completed

O No (answer all questions 5 thru 14)

Untreated decay

5. Untreated decay

O Yes O No

☐ Anterior(s)

☐ Premolar(s)

☐ Molar(s)

© Special Olympics, Inc. 2004
All rights reserved.

Definition
We define "Untreated decay" as at least ***one*** area of cavitation that would accommodate a 0.5 mm-diameter (or larger) bur or ball burnisher. **Observe all visible surfaces of the primary and permanent dentitions. Do not consider third molars.**

Untreated decay exists if on at least one tooth:

- cavitation is present that would accommodate no less than 0.5-millimeter-diameter bur or ball burnisher. If you are not sure cavitation exists, consider the tooth sound and mark "**No**";
- decay that fits the definition is present on any surface of the tooth (see photo #1), including root surfaces;
- root tips remain after severe caries has destroyed the rest of the tooth (see photo #2);
- there are restorations with recurrent decay fitting the definition of decay (see photo #3); or
- there are fractured, unrestored teeth with decay fitting the definition of decay (see photo #4).

TIP: In each of the last two examples, mark two categories: "untreated decay" and "filled teeth" for photo #3, and "untreated decay" and "injury" for photo #4.

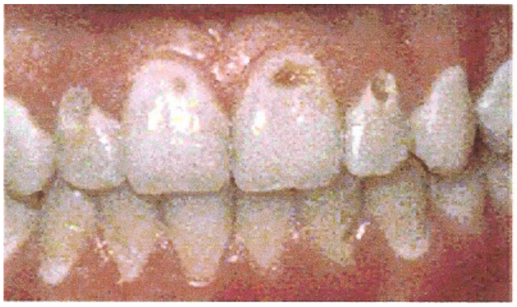

Photo #1

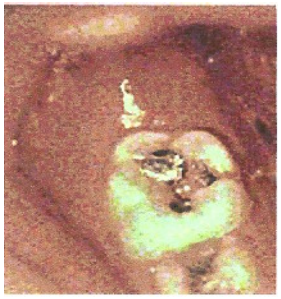

Photo #3

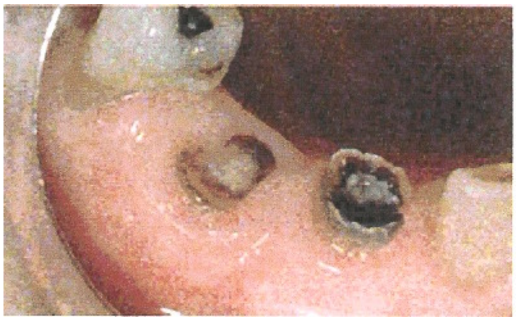

Photo #2

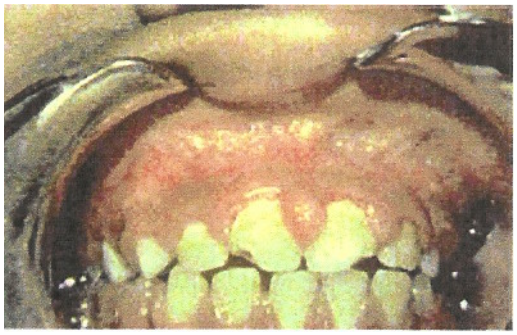

Photo #4

Mark "**Anterior(s)**" if untreated decay exists on central and/or lateral incisor(s) and/or cuspid(s). Otherwise leave blank.

Mark "**Premolar(s)**" if untreated decay exists in first or second premolar teeth. Otherwise leave blank.

Mark "**Molar(s)**" if untreated decay exists in first or second molar teeth (do not consider third molars). Otherwise leave blank.

© Special Olympics, Inc. 2004
All rights reserved.

The following are not considered decay. Mark “**No**” if:

- no teeth fit the definition of decay;
- cavitation is *less than* 0.5 millimeter in diameter;
- decalcification exists without cavitation (see photo #5);
- there are stained grooves without cavitation (see photo #6);
- fractured teeth are free of decay or have decay not fitting the definition (see photo #7); or
- missing fillings have no decay fitting the definition (then mark “Filled teeth” category only; see section on Filled Teeth).

If no cavitation exists or you are not sure, consider the tooth sound.

TIP: Do not automatically mark fractured teeth and/or lost or partially lost restorations as decay; mark decay only if cavitation fitting the definition is present alone or with other conditions.

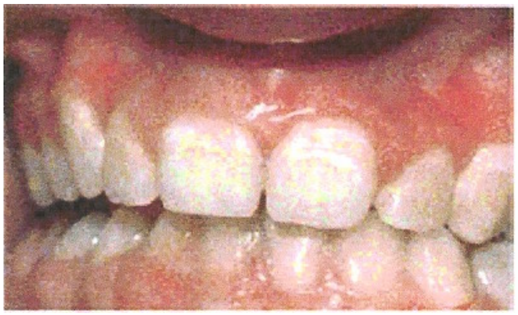

Photo #5

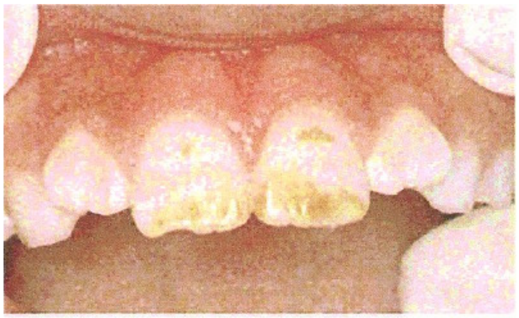

Photo # 6

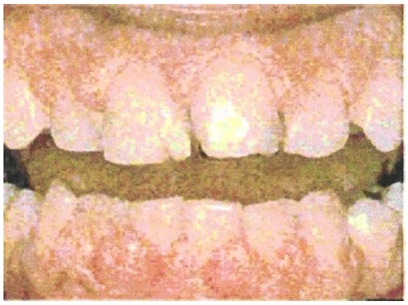

Photo #7

© Special Olympics, Inc. 2004
All rights reserved.

6. Filled teeth

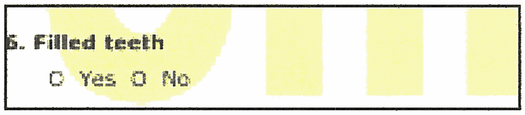
6. Filled teeth
○ Yes ○ No

Definition

We define "Filled teeth" as any dental work *done exclusively as a response to decay*. **Observe all surfaces of the primary and permanent dentitions. Do not consider third molars.**

Mark "**Yes**" if on at least one tooth there is:

- a restoration (e.g., fillings, inlays or crowns, including stainless steel crowns) of any type of material (including temporary materials);
- an interproximal restoration (see photo #8);
- a partially or entirely lost restoration. *IMPORTANT:* Do not automatically mark lost fillings as decay *unless* cavitation fitting the definition of decay is *also* present; then mark *both* "**Yes**" in the "Filled teeth" category and the appropriate item in the "Untreated decay" category (see section on Untreated Decay); or
- a lingual restoration (placed due to decay rather than due to root canal access) on an anterior tooth with or without other fillings, and no discoloration (see photo #9)

Mark "**No**" if:

- there are no restorations in the mouth;
- only anterior teeth are restored, with *incisal* restorations or crowns (see photo #10); or
- the only tooth under consideration is discolored (indicating loss of pulpal vitality) with a lingual restoration. Refer to the section on Injury.

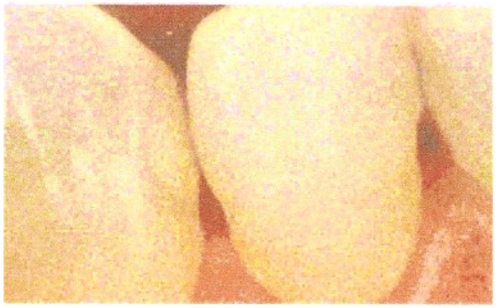

Photo #8

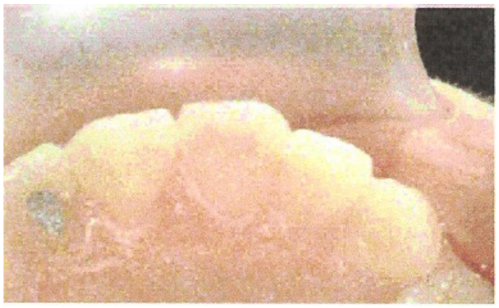

Photo #9

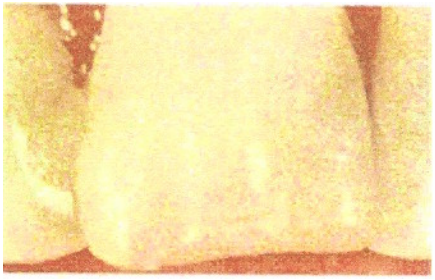

Photo #10

© Special Olympics, Inc. 2004
All rights reserved.

Special Considerations Associated with Anterior Teeth

- *Incisal* (diagonal or horizontal; see photo #10) restorations and/or crowns on anterior teeth *may or may not* be due to decay; therefore do not mark them as filled (refer to the section on Injury).
- *Interproximal* restorations are considered as placed due to decay; therefore consider them filled.
- Crowns on anterior teeth are not marked as filled.
- Cause of *lingual restorations on anterior* teeth will be assessed as follows:
    a) If the anterior tooth with a lingual restoration has other fillings, consider the tooth decayed and mark as filled.
    b) If the anterior tooth with a lingual restoration also has discoloration, do not consider the tooth decayed and do not mark it as filled (instead, consider the tooth when completing the cycle for the “Injury” category — refer to the section on Injury).
    c) If the anterior tooth with a lingual restoration has no other fillings and no discoloration, consider the tooth decayed, and mark it as filled (see photo #11).

TIP: Is there another restoration fitting the definition that would eliminate the need to evaluate fillings on anterior teeth? Remember, you are looking for one or more teeth that fit the definition.

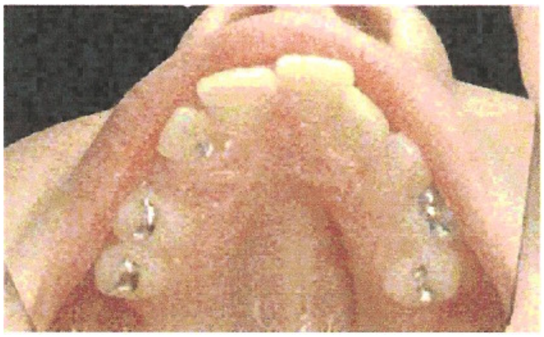

Photo #11

7. Missing Teeth

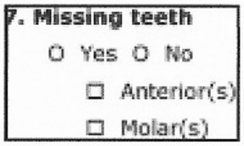

7. Missing teeth

O Yes O No

☐ Anterior(s)

☐ Molar(s)

Definition

Any tooth not present at the time of the exam is considered missing subject to the following rules: Unerupted teeth are not counted as missing (an eruption chart is included on page 19 for your convenience). **Consider only the permanent dentition.** We define **“Anterior(s)” as central incisors, lateral incisors and cuspids.** We define **“Molar(s)” as first and second molars. Do not consider premolars or third molars.**

© Special Olympics, Inc. 2004
All rights reserved.

Mark "**Yes**" and "**Anterior(s)"** if:

- one or more permanent central incisors, lateral incisors or cuspids that fit the definition of missing teeth are missing; or
- missing teeth that fit the definition have been replaced with prosthetics.

Mark "**Yes**" and "**Molar(s)**" if:

- one or more permanent first or second molars that fit the definition of missing teeth are missing;
- missing teeth that fit the definition have been replaced with prosthetics.

Mark "**No**" if:

- no teeth are missing; or
- the only teeth missing are premolars and/or third molars (see photo #13).

TIP: You cannot mark either "Anterior(s)" or "Molar(s)" and "No." You *can* mark both "Anterior(s)" and "Molar(s)."

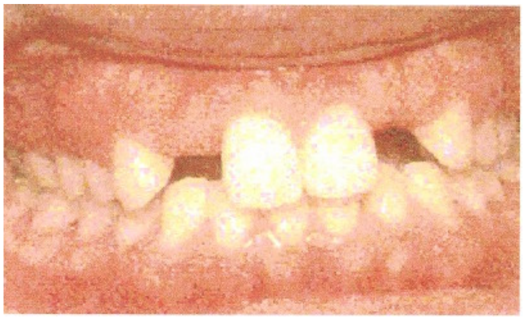

Photo #12

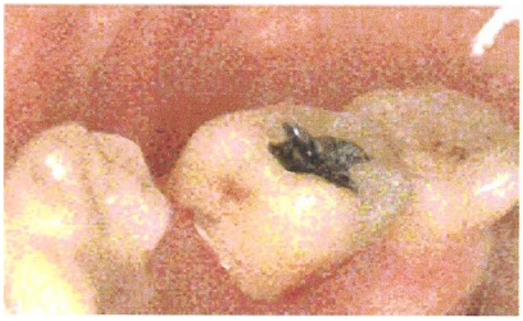

Photo #13

© Special Olympics, Inc. 2004
All rights reserved.

Eruption Dates for Use in Determining Missing Teeth

Anterior Teeth

- Permanent **maxillary central incisors** should be in place after 9 years of age.
- Permanent **mandibular central incisors** should be in place after 7 years of age.
- Permanent **maxillary lateral incisors** should be in place after 10 years of age.
- Permanent **mandibular lateral incisors** should be in place after 8 ¾ years of age.
- Permanent **maxillary cuspids** should be in place after 13 years of age.
- Permanent **mandibular cuspids** should be in place after 12 years of age.

Posterior Teeth

- Permanent **first molars** should be in place after 7 years of age.
- Permanent **second molars** should be in place after 13 years of age.

Adapted from Finn SB. Clinical pedodontics. Philadelphia: W.B. Saunders Company, 1973

© Special Olympics, Inc. 2004
All rights reserved.

8. Sealants

8. Sealant(s)
O Yes O No

We define "sealant(s)" as material placed as a preventive measure, **covering the occlusal surface(s) of first and/or second molars. Consider only the permanent dentition.**

Mark "**Yes**" if on at least one tooth:

- the occlusal surface of a permanent first or second molar has been sealed (see photo #15); or
- any part of the sealant remains covering the surface.

Mark "**No**" if:

- a preparation appears to have been cut for the placement of filling material (see photo #15);
- sealants are present on other teeth, but not on the occlusal surfaces of the first and second molars; or
- you are not sure that there is sealant material on the tooth.

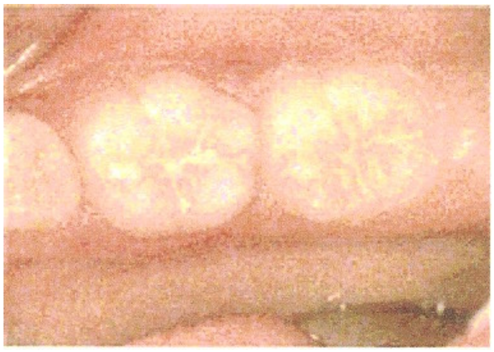

Photo #14

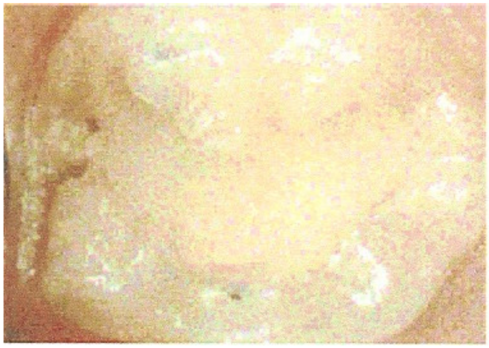

Photo #15

9. Injury

9. Injury
O Yes O No

Definition
Consider only maxillary and mandibular central and lateral incisors in the permanent dentition. Do not consider primary teeth.

© Special Olympics, Inc. 2004
All rights reserved.

Mark “**Yes**” if a tooth fitting the definition is:

- missing (missing homologous teeth are **not** considered to be due to an injury) (see photo #16);
- discolored (indicating no pulpal vitality) and sound (see lower central incisor, photo #17);
- discolored and has a lingual restoration (see section on Filled teeth);
- fractured with or without decay (see upper central incisors, photo #18); or
- fractured with or without a restoration (see upper central incisors, photo #18).

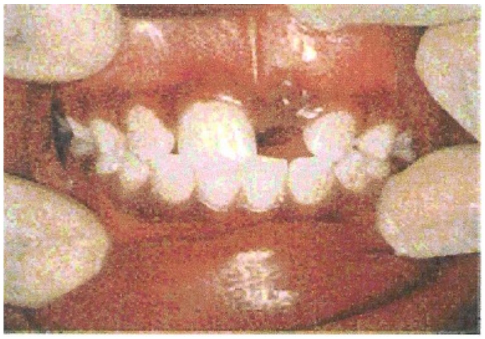

Photo #16

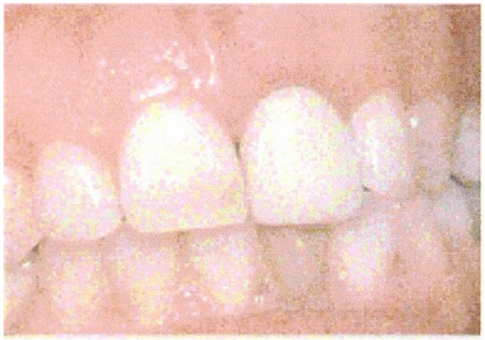

Photo #17

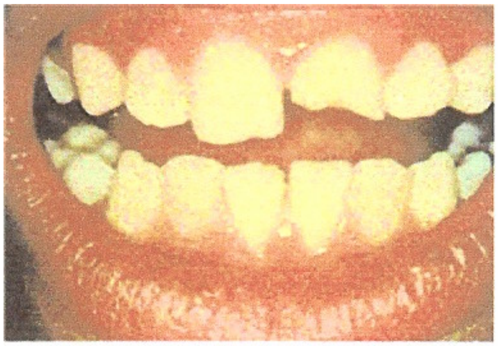

Photo #18

Mark "**No**" if there is:

- no sign of injury to central and/or lateral incisors;
- homologous teeth are missing;
- only an interproximal restoration, suggesting decay rather than injury (see photo #19);
- a crown on a central and/or lateral incisor (see photo #20); or
- injury to teeth *other than* central and lateral incisors.

TIP: Crowns on anterior teeth are not marked in "Filled teeth” or "Injury” categories because there is no way to determine if the crown was placed due to decay or injury. **Do not mark *anterior crowns* in any category.** You should, however, mark interproximal restorations (as *filled*) and horizontal or vertical restorations (as *injury*) *on anterior teeth*.

© Special Olympics, Inc. 2004
All rights reserved.

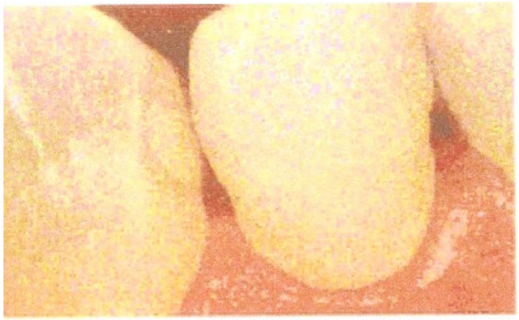

Photo #19

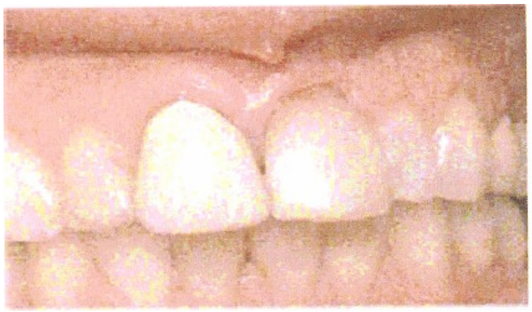

Photo #20

10. Fluorosis

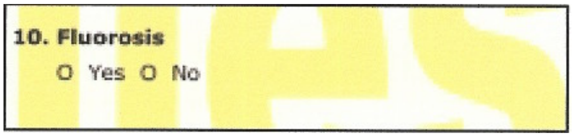

10. Fluorosis
O Yes O No

Definition
We define "Fluorosis" as a condition ranging from small, diffuse, opaque, paper-white areas to the presence of brown stains and pitting scattered over *at least* 25 percent of the tooth surface. **Observe only the buccal area of the maxillary arch, cuspid to cuspid. Consider only the permanent dentition.**

Mark "**Yes**" if:

- fluorosis (as defined) occurs on homologous teeth specified (for example, two central incisors, two lateral incisors, and/or two cuspids; see photo sequence #21, #22, #23 and #24).

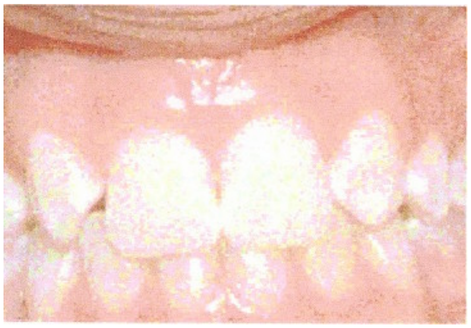

Photo #21

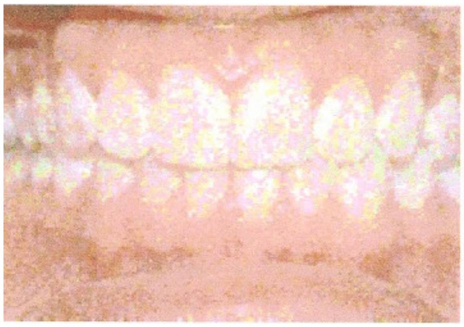

Photo #22

© Special Olympics, Inc. 2004
All rights reserved.

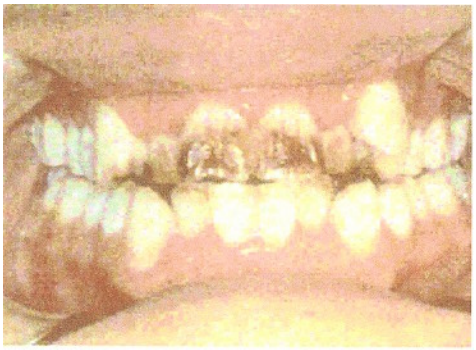

Photo #23

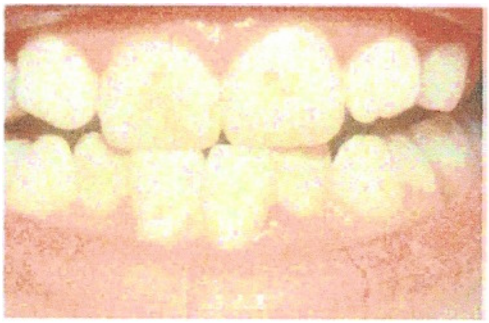

Photo #24

Mark “**No**” if:

- the differential diagnosis (see Russell’s table on page 24) suggests a condition other than fluorosis (see photo #25);
- only one tooth is affected, rather than homologous teeth (see photo #25);
- less than 25 percent of teeth considered show fluorosis fitting the definition (photo #26); or
- teeth are partially erupted (have not reached occlusion).

Note: Although mandibular teeth are displayed in the photos below, they are **not** assessed in determining fluorosis.

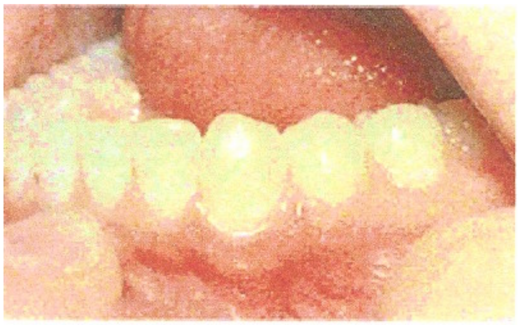

Photo #25

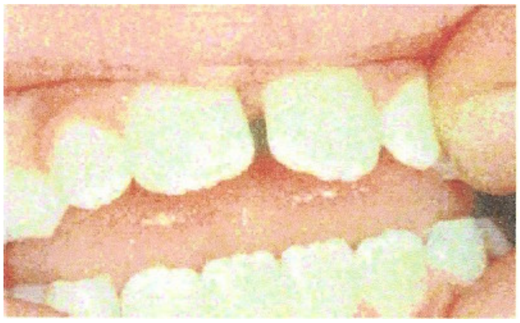

Photo #26

© Special Olympics, Inc. 2004
All rights reserved.

The Differential Diagnosis of Fluoride and Non-Fluoride Enamel Opacities

Characteristic	Milder forms of Fluorosis	Non-Fluoride Opacities
Area affected	Usually seen on or near cusp tips or incisal edges	Usually centered on smooth surface; may affect entire crown
Shape of lesion	Resembles line shading in pencil sketch; lines follow incremental lines in enamel and form irregular caps or cusps	Often round or oval
Demarcation	Shades off imperceptibly into surrounding normal enamel	Clearly differentiated from adjacent normal enamel
Color	Slightly more opaque than normal enamel; "paper–white." Incisal edges, tips of cusps may have frosted appearance. Does not show stain at time of eruption (in these milder degrees, rarely at any time)	Usually pigmented at the time of eruption; often creamy-yellow to dark reddish-orange
Gross hypoplasia	None. Pitting of enamel does not occur in the milder forms. Enamel surface has glazed appearance and is smooth to an explorer	Absent to severe. Enamel surface may seem etched or rough to an explorer
Detection	Often visible under strong light; most easily detected by line of sight tangential to the crown	Seen most easily under strong light on line of sight perpendicular to tooth surface.

Source: Adapted from Russell AL. The differential diagnosis of fluoride and non-fluoride enamel opacies. J. Public Health Dent 1961; 21:143-6.

© Special Olympics, Inc. 2004
All rights reserved.

11. Gingival signs

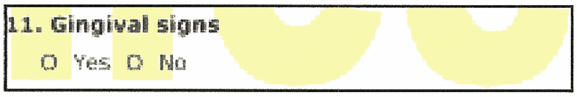
11. Gingival signs

O Yes O No

Definition

We define “Gingival signs” as free or attached gingival margins or papillae that are moderately red, or show significant deviations from “normal” contour or texture, occurring on the gingiva of three or more teeth within the specified area. **Observe only the buccal area of the mandibular arch, cuspid to cuspid. Consider only the permanent dentition.**

Mark “**Yes**” if:

- buccal gingiva on three or more teeth in specified area are at least moderately to severely red or blue (see photo #27 and #28);
- buccal gingiva on three or more teeth in specified area show, for example, loss of stippling; or
- glossiness, fibrosis, frank swelling or enlargement, hyperplasia, and/or flaccidity of attached gingiva (see photo #28).

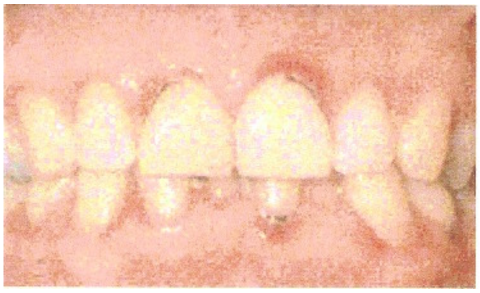

Photo #27

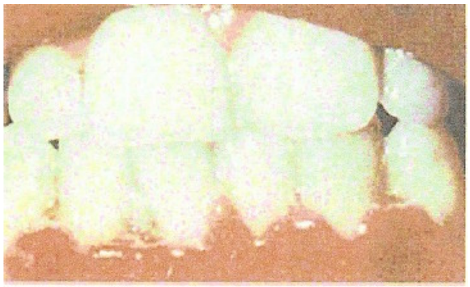

Photo #28

Mark “**No**” if:

- buccal gingiva on fewer teeth than three teeth in specified area are affected;
- gingiva demonstrates only slight redness or change in contour or texture (see photo #29);
- gingival signs occur on teeth other than the buccal area of the mandibular arch, cuspid to cuspid;
- a recent surgical procedure within the defined area is obvious;
- recent injury within the defined area is obvious; or
- you are in doubt whether gingival signs are present.

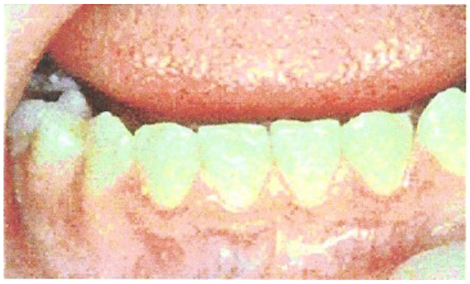

Photo #29

© Special Olympics, Inc. 2004
All rights reserved.

1.2. Treatment urgency

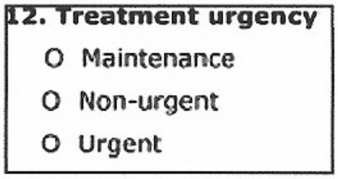
12. Treatment urgency
O Maintenance
O Non-urgent
O Urgent

Definition
Treatment urgency is based upon findings under the sections on Dental History and Screening.

Mark "**Maintenance**" if there is no pain inside the mouth, and:
- no untreated decay;
- no untreated injuries to the teeth; or
- you marked "no" for gingival signs.

Mark "**Non-urgent**" (see photo #30) if there is no pain inside the mouth, and:
- decay not involving the pulp;
- broken fillings without decay; or
- you marked "yes" for gingival signs *and* there are no abscesses present.

Mark "**Urgent**" (see photo #31) if any or all of the following are present:
- any pain inside the mouth;
- possible pulpal involvement (whether due to decay or injury);
- broken or missing fillings with decay; or
- periodontal abscesses are present.

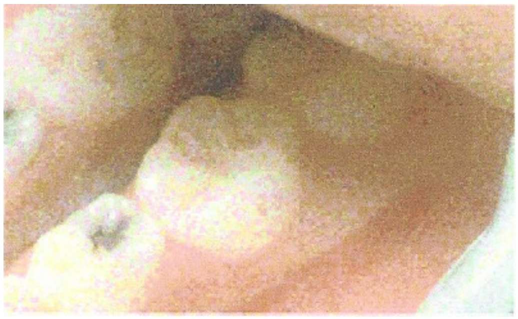
Photo #30

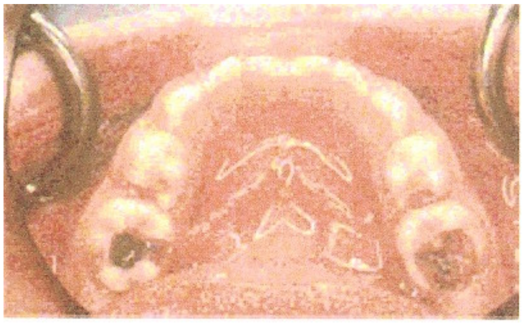
Photo #31

© Special Olympics, Inc. 2004
All rights reserved.

13. Mouth guard recommended

13. Mouth guard recommended
○ Yes ○ No
□ Mouth guard delivered

Definition
Check or ask the athlete what sport they participate in. If it is a contact sport such as basketball, mark "**Yes**."

14. Exam Complete

14. □ Exam Complete

Definition
At end of the exam, mark exam complete.

© Special Olympics, Inc. 2004
All rights reserved.

Special Olympics

Healthy Athletes
Special Smiles®

Procedures for Screeners

A. The **screener** and the recorder are ready at their station. The **screener** is prepared with disposable gloves, a disposable mouth mirror, a disposable mask and a flashlight used to help in viewing the teeth. Fresh batteries will be in the flashlight, and replacement batteries will be provided as necessary to maintain a consistently bright beam. The recorder has an unmarked Special Smiles Screening Form and Athlete Dental Report Card.

B. The athlete arrives and gives his/her Screening Form to the recorder.

C. Each site will give instructions on who (i.e., persons at the registration desk, recorder) will fill in the categories: First name, Last name, HAS ID, Date, Sex, Date of Birth, Age and location information as required by the site.

D. The **screener** and the recorder begin by asking the questions in the Dental History section.

E. Before assessing categories under “**Screening**,” the screener must ask the athlete, **“Can I look in your mouth today?”** If the athlete says “**Yes**,” then the screening continues. If the athlete says “No,” then the recorder marks the **No. #3, “Athlete refused/could not screen,”** box on the form, *keeps the form* and directs the athlete to the next station. The athlete may exit the screening process at any time.

F. The recorder now prompts the **screener** through the Screening section; it is important that the **screener** follows the sequence below for each athlete.

Please Note: These conditions are defined on the following pages.

4. Edentulous
5. Untreated decay
6. Filled teeth
7. Missing teeth
8. Sealant(s)
9. Injury
10. Fluorosis
11. Gingival signs
12. Treatment urgency
13. Mouth guard recommended
14. Exam completed

© Special Olympics, Inc. 2004
All rights reserved.

4. **Edentulous**
The **screener** begins by checking for edentulism. If the athlete is edentulous, the screening process is completed; the recorder marks “**Yes**” and “**Exam completed**” on the screening form and the Dental Report Card, and the athlete is directed to the next station. If there are teeth or root tips present, the screening continues.

5. **Untreated decay**
In this first cycle, the **screener** considers the **primary and permanent dentitions starting with the anterior teeth, and proceeding to the premolars and then the molars.** The recorder marks each and all categories that have at least one tooth fitting the definition of decay. If, by the completion of this cycle, no teeth have untreated decay fitting the case definition, the recorder marks “**No**.” *Note that if any or all of the categories that have at least one tooth fitting the definition of "decay" are marked, then “**No**” cannot be marked as well.*

6. **Filled teeth**
The screener considers both **the primary and permanent dentitions, beginning with the upper-right quadrant, proceeding to the upper-left and then the lower-left quadrant, and finishing with the lower-right quadrant, examining all surfaces of the teeth.** This second cycle progresses until the first tooth is seen fitting the case definition of filled teeth; the recorder marks “**Yes**.” If there are no filled teeth fitting the case definition by the completion of this cycle, the recorder marks “**No**.”

In the next five cycles, **the screener** considers only the **permanent dentitions**.

7. **Missing teeth**
The third cycle progresses through the **maxillary and then the mandibular arch, cuspid to cuspid, and proceeds to the maxillary and then the mandibular first and second molars. Premolars are not considered.** The recorder marks missing teeth that fit the case definition as “**Anterior(s)**” and/or “**Molar(s)**.” If there are no teeth fitting the case definition at the completion of this cycle, the recorder marks “**No**.”

8. **Sealants**
The fourth cycle begins with the upper-right and then the upper-left first and second molars, and proceeds to the lower-left and then the lower-right first and second molars. If a sealant fitting the case definition is present, the recorder marks “**Yes**.” If there are no sealants present at the completion of this cycle, the recorder marks “**No**.”

Recent unpublished data suggest that the use of toothpicks increases the sensitivity (the probability of a sealant being detected if it is present) of detecting sealants. Since the use of toothpicks implies potential contact of the **dental screener’s** hand with oral tissues, gloves should be used with the use of toothpicks.

9. **Injury**
The sixth cycle progresses through the maxillary and then the mandibular central and lateral incisors. If the dental screener sees injury that fits the case definition, the recorder marks “**Yes**.” If there is no injury fitting the case definition at the completion of this cycle, the recorder marks “**No**.”

© Special Olympics, Inc. 2004
All rights reserved.

10. **Fluorosis**
The sixth cycle progresses through the maxillary arch, cuspid to cuspid, buccal surfaces only. Homologous teeth must be affected by fluorosis for “**Yes**” to be marked. If there is no fluorosis fitting the case definition at the completion of this cycle, the recorder marks “**No**.”

11. **Gingival signs**
We define “Gingival signs” as free or attached gingival margins or papillae that are moderately red, or show significant diviations from “normal” contour or texture, occuring on the gingiva of three or more teeth within the specified area. **Observe only the buccal area of the mandibular arch, cuspid to suspid. Consider on the permanent dentition.** Three or more teeth must fit the case definition of gingival signs for “**Yes**” to be marked. If there are no gingival signs fitting the case definition at the completion of this cycle, the recorder marks “**No**.”

12. **Treatment urgency**
The eighth and final cycle is a full cycle. The screener considers both the permanent and primary dentitions and the soft tissues of the oral cavity and gives a response of “**Maintenance**” “**Non–urgent**,” or “**Urgent**” to the recorder on the basis of the overall condition of the mouth. The recorder fills out “**Treatment urgency**” on the Screening Form and on the Dental Report Card, and marks No. 14, “**Exam Completed**,” on the Screening Form. The Screening Form is retained and the recorder gives the Dental Report Card to the athlete and directs him/her to the next station or exit.

13. **Mouth guard recommended**
Check or ask the athlete what sport they participate in. If it is a contact sport such as basketball, mark “**Yes**.”

14. **Exam completed**
At the end of the exam, mark “**Exam completed**.”

G. The **dental screener** disposes of the mouth mirror, mask, and gloves in the predetermined manner.

Please refer to the next page for a summary chart on the procedure.

© Special Olympics, Inc. 2004
All rights reserved.

Special Olympics

Healthy Athletes Special Smiles®

Screening Procedures Summary Chart

Note: To help guide your screeners during the event, you may want to copy this chart (and laminate it, if possible) and have it available for ready reference.

5. Untreated decay	6. Filled teeth	7. Missing teeth	8. Sealant(s)	9. Injury	10. Fluorosis	11. Gingival signs	12. Treatment urgency
DENTITION							
Primary & permanent	Primary & permanent	Permanent	Permanent	Permanent	Permanent	Permanent	Primary & permanent
SEQUENCE							
Maxillary, then mandibular; anteriors to premolars to molars	Full cycle starting with upper-right quadrant	1. Maxillary cuspid to cuspid<br>2. Mandibular cuspid to cuspid<br>3. Maxillary molars<br>4. Mandibular molars	First and second molars starting with upper-right quadrant	1. Maxillary central & lateral incisors<br>2. Mandibular central & lateral incisors	Maxillary cuspid to cuspid	Mandibular cuspid to cuspid	Full cycle starting with upper-right quadrant
SURFACES							
All	All	N/A	Occlusal	N/A	Buccal	Buccal	* All tooth surface<br>* All soft tissue

Guidelines:

- Assess each condition in a separate cycle, independent of all other conditions.
- If two conditions are present on the same tooth, both are marked.
- Consider all visible surfaces on orthodontically banded teeth.
- Do not consider third molars or partially erupted teeth (teeth not in occlusion

© Special Olympics, Inc. 2004
All rights reserved.

Objectives for Recorders

At the completion of training, the **recorder** will be able to:

1.) Understand the importance of accuracy in collecting information and recording screening;

2.) Prompt the dental screener through the correct sequence on the Screening Form; and

3.) Accurately complete a Screening Form for each athlete.

© Special Olympics, Inc. 2004
All rights reserved.

Special Olympics

Healthy Athletes Special Smiles®

Screening Procedure for Recorders

Please take a moment to review the following procedures that you, as a **recorder**, are responsible for during the oral health screening for Special Olympics athletes. Please note that these procedures may differ slightly at each site.

A. The screener and the **recorder** are ready at their station. The **recorder** has readily available: disposable gloves, mouth mirrors, masks and toothpicks for the screener;

- replacement batteries for the flashlight (replace the flashlight and/or batteries with a frequency that allows a consistently bright beam);
- an unmarked Dental Report Card; and
- pens or pencils.

B. The athlete arrives and gives his/her Screening Form to the **recorder**.

C. The site will determine if the **recorders** fill out portions of the Event Specifics and Athlete Demographics sections. The screener and **recorder** will *always fill* in the "examiner" top half of the form and the section on Dental History and Screening.

D. The screener and **recorder** begin by asking the questions in the Dental History section.

E. Before assessing categories under "Screening," the screener must ask the athlete, "Can I look at your teeth today?" If the athlete says "yes," then the screening continues. If the athlete says "no," then the **recorder** marks **No. 3**, "**Athlete refused/could not screen**," on the form, *keeps the form* and directs the athlete to the next station. The athlete may exit the screening process at any time.

F. It is the responsibility of the **recorder** to prompt the screener through the section on Screening *specifically in the sequence listed* and to record the responses given by the screener. This will allow the screener to do visual cycles in logical order.

Questions:

4. Edentulous
5. Untreated decay
6. Filled teeth
7. Missing teeth
8. Sealant(s)
9. Injury
10. Fluorosis
11. Gingival signs
12. Treatment urgency
13. Mouth guard recommended
14. Exam completed

© Special Olympics, Inc. 2004
All rights reserved.

G. At the completion of the screening, the **recorder**:

- ensures that all sections on the Screening Form are completed;
- marks **No. 14**, "**Exam Completed**," on the form;
- *IMPORTANT:* retains and stores the Screening Form in the predetermined place;
- marks the Dental Report Card with the same response under the "Treatment urgency" category on the Screening Form (the wording on the Dental Report Card may differ slightly from the wording on the Screening Form); and
- gives the Dental Report Card to the athlete and directs the athlete to the next station or exit, as appropriate.

© Special Olympics, Inc. 2004
All rights reserved.

Tips for Recorders Using Screening Forms

Thank you for your time and help in collecting information on the oral health status of Special Olympics athletes! Your efforts now in quality assurance will be very helpful after the event in ensuring complete information and in analyzing the data. These analyzed data will be used by Special Olympics Special Smiles to learn more about the oral health status of athletes and, based on the findings, to direct advocacy efforts for people with special needs.

Following are some useful guidelines and suggestions to help you fill out the Screening Form:

- It is **very important** that you collect and retain a Screening Form from each and every athlete, even if the person refused or could not be screened. Before starting to screen the athletes, know the designated place to store the completed forms.
- Fill in the Screener's name or ID number.
- Coaches may be screened as a courtesy if they request it, but *do not fill* out Screening Forms for anyone other than Special Olympics athletes. Athletes are the only group that data are being collected on at this time.
- You are the last person to see the Screening Form while the athlete is still present; therefore it is your responsibility to scan the form and be sure that all areas are completed, *even if some areas on the form are assigned to be filled out by others at the site*. The exception is the Screening section, left blank *only* if the athlete was edentulous or refused the screening. To identify these cases, you should fill out the **No. 3**, "**Athlete refused/could not screen**," box, or on **No. 4**, **Edentulous**, mark "**Yes**."

© Special Olympics, Inc. 2004
All rights reserved.

Standardization Exercise

For this exercise, base all decisions on the case definitions in the manual. Refer to the Standardization Exercise photos on page 38 to answer the questions.

Photo A

1. You are doing a cycle for Untreated decay. If you were considering only the teeth shown, how would you mark this category?

5. Untreated decay
- O Yes O No
  - ☐ Anterior(s)
  - ☐ Premolar(s)
  - ☐ Molar(s)

Photo B

2. You are doing a cycle for Untreated decay. If you were considering only the teeth shown, how would you mark this category?

5. Untreated decay
- O Yes O No
  - ☐ Anterior(s)
  - ☐ Premolar(s)
  - ☐ Molar(s)

Photo C

3. Consider only the first molar, and assume that the void represents only a missing filling and not a cavitation. What category or categories would you mark for this tooth?

5. Untreated decay
- O Yes O No
  - ☐ Anterior(s)
  - ☐ Premolar(s)
  - ☐ Molar(s)

6. Filled teeth
- O Yes O No

Photo D

4. Consider only the first molar. What category or categories would you mark for this tooth?

5. Untreated decay
- O Yes O No
  - ☐ Anterior(s)
  - ☐ Premolar(s)
  - ☐ Molar(s)

6. Filled teeth
- O Yes O No

© Special Olympics, Inc. 2004
All rights reserved.

Special Olympics

Healthy Athletes Special Smiles®

PRACTICE TEST

Photo E
5. You are doing a cycle for Filled teeth. How would you mark this category?

6. Filled teeth
O Yes O No

Photo F
6. You are doing a cycle for Missing teeth. How would you mark this category?

7. Missing teeth
O Yes O No
☐ Anterior(s)
☐ Molar(s)

Photo G
7. You are doing a cycle for Filled teeth, and then for Injury. If you were considering only the right central incisor, how would you mark these categories?

6. Filled teeth
O Yes O No

9. Injury
O Yes O No

Photo H
8. You are doing a cycle for Fluorosis. How would you mark this category?

10. Fluorosis
O Yes O No

Photo I
9. You are doing a cycle for Gingival signs. How would you mark this category?

11. Gingival signs
O Yes O No

Photo J
10. You are doing a cycle for Treatment urgency. What level of urgency would you assign for this athlete?

12. Treatment urgency
○ Maintenance
○ Non-urgent
○ Urgent

© Special Olympics, Inc. 2004
All rights reserved.

11. You are looking in the athlete's mouth and note that he/she is edentulous. After marking the Edentulous category "yes," what do you do?

- Thank the athlete, and direct him/her to leave the screening area.
- Assist Treatment urgency.
- Assess Gingival signs.
- Mark #14, "Exam completed," on the form and direct the athlete to the next station in the screening room.

Once you have completed the exercise, please refer to page 39 to compare your responses with the answers found there.

© Special Olympics, Inc. 2004
All rights reserved.

Special Olympics

Healthy Athletes Special Smiles®

Standardization Exercise Photos

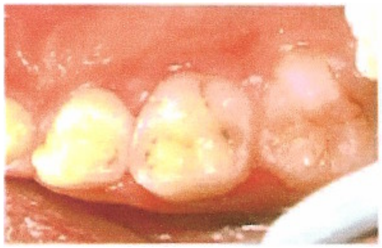
Photo A

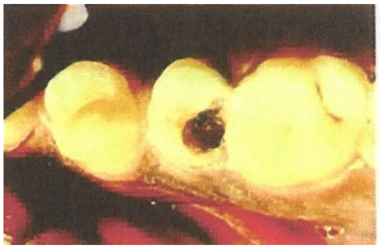
Photo B

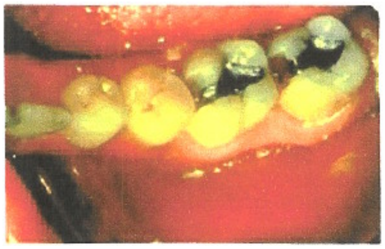
Photo C

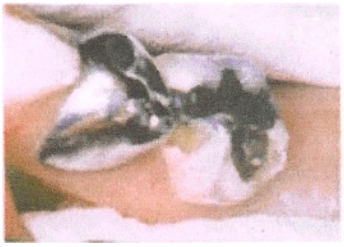
Photo D

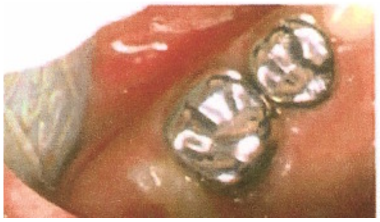
Photo D

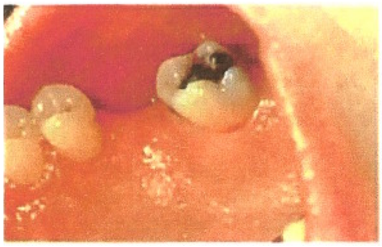
Photo F

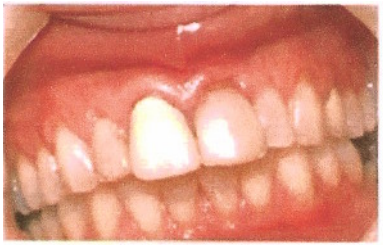
Photo G

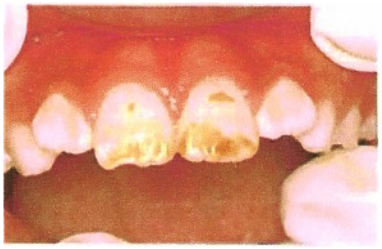
Photo H

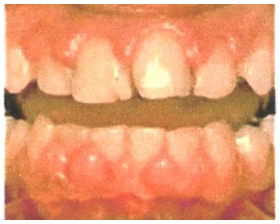
Photo I

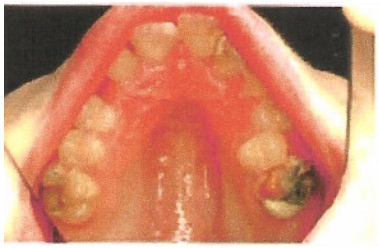
Photo J

© Special Olympics, Inc. 2004
All rights reserved.

Standardization Exercise Answers

Use these answers to compare with your responses to the standardization exercise that begins on page 30.

1. No
2. Premolar
3. No, Yes
4. No, Yes
5. Yes
6. Molar
7. No, No
8. Yes
9. Yes
10. Urgent
11. Mark #14, “Exam completed”

© Special Olympics, Inc. 2004
All rights reserved.

Special Olympics

Healthy Athletes
Special Smiles®

Non-Clinical List of Instructions for Training Sessions

1. Open your boxes of materials from Special Olympics immediately upon receipt to ensure you have all the materials you need; this will give you time to secure the missing items before the day of the event.

2. Keep all training materials for the following year, with the exception of the unused HAS forms. Unused forms should be returned with the used forms; however, they should be bundled separately.

3. As the Data Coordinator, please check or assign the responsibility of checking each form to see that all appropriate circles are filled in before returning the forms. While this job is primarily the responsibility of the recorder, experience has shown that he/she may be too busy on the day of the event to ensure this.

4. As the Data Coordinator, please check or assign the responsibility of making sure that only completed forms are counted and returned. Do not count or include forms that have had only the date and site number filled out in anticipation of screening more athletes.

5.) Count the completed forms. Write the total on a piece of paper (or use the sample on page X) and attach it to the top of the forms to be sent. Also include on this sheet the number of screeners trained throughout the day.

6.) Send the bundle of completed forms, with the cover sheet on top, to Shantae Polk, Manager, Health and Research Initiatives, at Special Olympics headquarters.

© Special Olympics, Inc. 2004
All rights reserved.

Cover Sheet for Completed HAS Forms

Copy this page and use it as a cover sheet for the completed HAS forms you will send to Special Olympics.

Special Olympics Special Smiles Site Name: ____

City: ____

State: ____

Date(s) of Special Smiles event (m/d/y): ___ ___ ___

No. of completed HAS forms: _____________

No. of screeners trained: _________________

Unused forms enclosed: ______Yes ______No

Name of Site Data Coordinator: ____

© Special Olympics, Inc. 2004
All rights reserved.

Special Smiles Contact Information

Steven Perlman
Special Smiles Global Clinical Advisor
c/o Dr. Perlman & Koidin, PC
77 Broad St.
Lynn, MA 01902
+1 (781) 599-2900
+1 (781) 598-1670 (Fax)
sperlman@bu.edu

Shantae L Polk
Manager, Health and Research Initiatives
Special Olympics Special Smiles & FUNfitness
+1 (202) 824-0239
+1 (202) 824-0397 (Fax)
spolk@specialolympics.org

Dr. Mark Wagner
Director, Health & Research Initiatives
+1 (202) 715-1148
mwagner@specialolympics.org

Kristen Malina
Research Assistant
+1 (202) 715-3600
kmalina@specialolympics.org

© Special Olympics, Inc. 2004
All rights reserved.

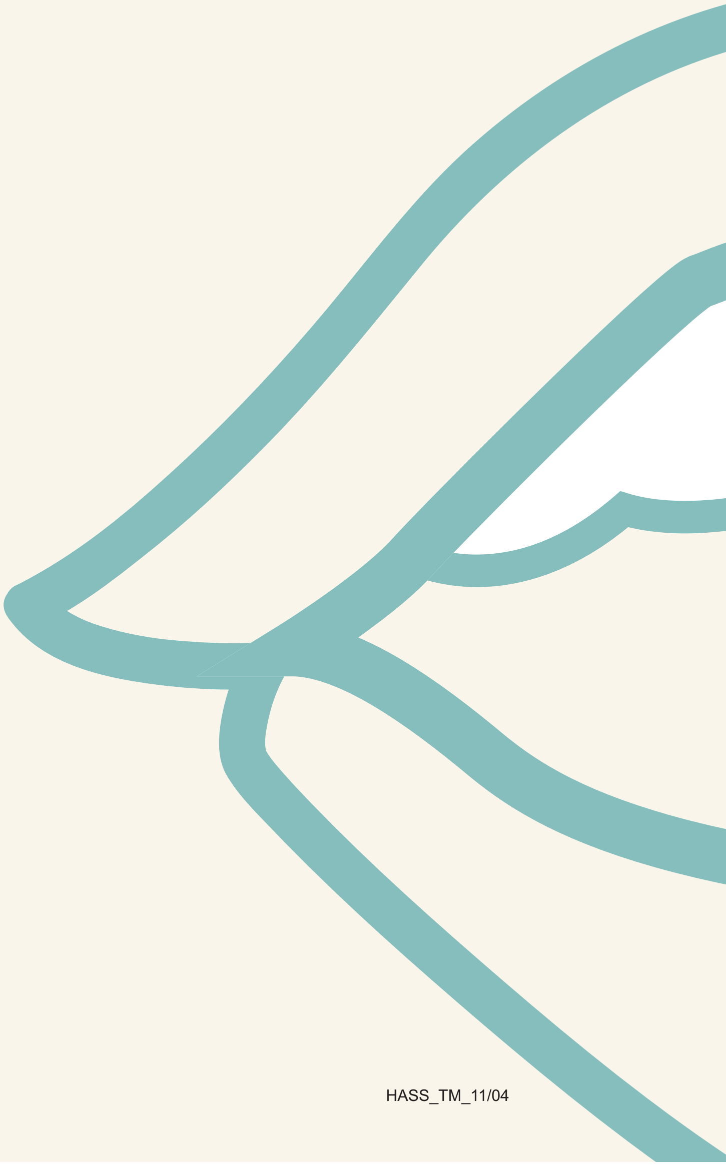

Special Olympics

www.specialolympics.org

Created by the Joseph P. Kennedy, Jr. Foundation for the Benefit of Persons with Intellectual Disabilities

HASS_TM_11/04
