# Supplementary material for: Oral health of individuals with intellectual disabilities: a global cross-sectional study from the special olympics world games 2023
Source: Clin Oral Investig. 2025 Apr 16;29(5):252. doi: 10.1007/s00784-025-06331-3 (PMC12003447; doi:10.1007/s00784-025-06331-3)

Supplements:

Supplementary Figure 1 a+b: Standardized oral health screening during the Special Olympics World Games Berlin 2023.


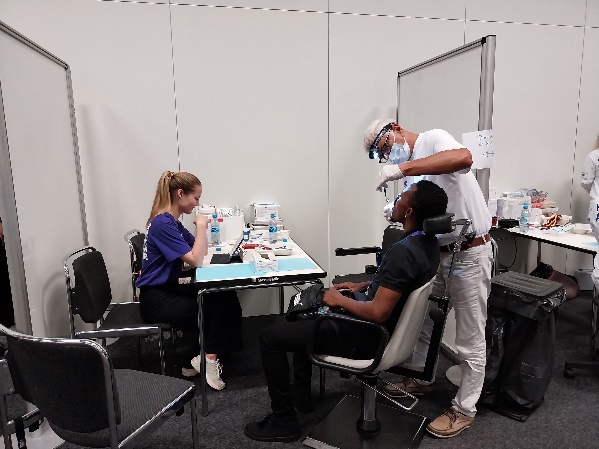

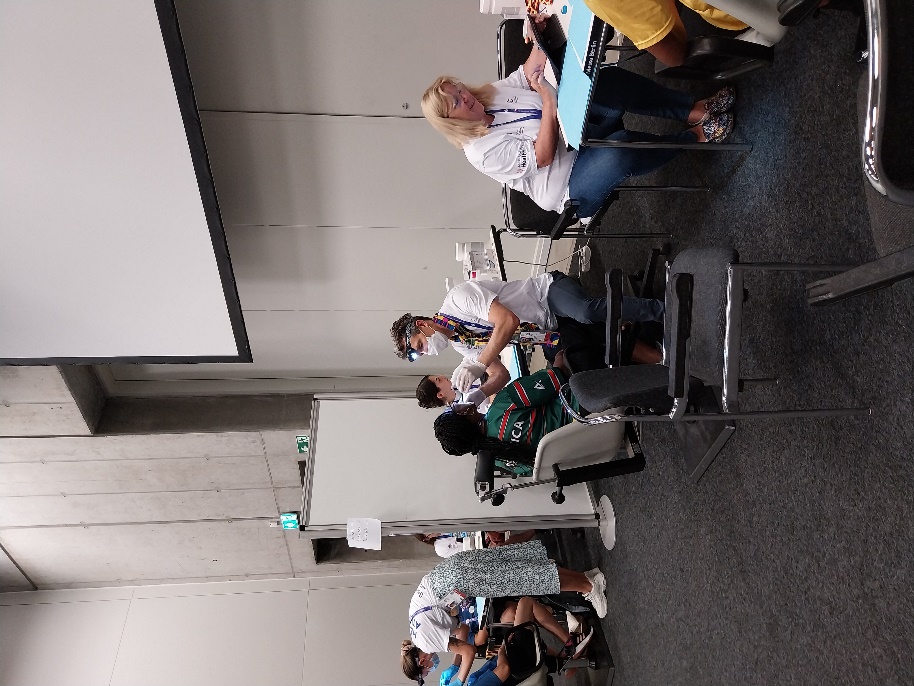


Supplementary Figure 2: Distribution of sports
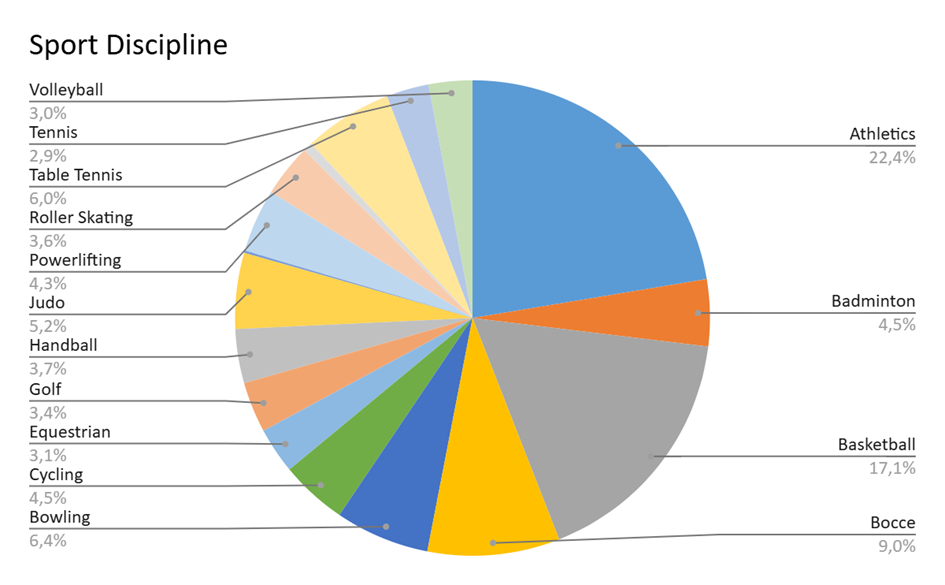

Supplement: Supplementary file 2 — Supplementary Material 2 [file 784_2025_6331_MOESM2_ESM.docx]
